# Fatty Acid-Binding Protein 3 (*FABP3*)-Mediated Abdominal Fat Deposition in Broilers via the PPAR Signaling Pathway

**DOI:** 10.3390/ijms27219413

**Published:** 2026-10-22

**Authors:** Hui Yuan, Yuxin Zhu, Kaibo Jin, Yuan Liu, Jun Lu, Runxiang Zhang

**Affiliations:** College of Animal Science and Technology, Northeast Agricultural University, Harbin 150030, China; huiyuan@neau.edu.cn (H.Y.); ttkx632278418@163.com (Y.Z.); 18545627620@163.com (K.J.); 18846816101@163.com (Y.L.); 18800425725@163.com (J.L.)

**Keywords:** broilers, abdominal fat deposition, meta-analysis, FABP3, PPAR pathway

## Abstract

Excessive deposition of abdominal fat in broilers compromises feed efficiency, carcass yield, processing efficiency, and inflates production costs. Because this trait is governed by multi-tissue crosstalk coupling hepatic lipid synthesis to adipose lipid storage, its coordinated regulation has seldom been addressed. We combined three abdominal fat transcriptomic datasets (GSE42980, GSE49121, GSE104039) with one hepatic dataset (GSE104038), obtaining 2520 meta-differentially expressed genes (meta-DEGs). Functional enrichment revealed a prominent enrichment of lipid-metabolism terms, including peroxisome proliferator-activated receptor (PPAR) signaling. Fatty acid-binding protein 3 (*FABP3*) appeared repeatedly within the Gene Ontology (GO), Kyoto Encyclopedia of Genes and Genomes (KEGG) and gene set enrichment analysis (GSEA) lipid-metabolism categories and was therefore taken forward for functional work. Overexpressing *FABP3* enhanced proliferation, lipid accumulation and lipogenic marker expression, whereas knockdown reversed these effects. In both adipocytes and hepatocytes, *FABP3* raised peroxisome proliferator-activated receptor gamma (*PPARγ*) and fatty acid-binding protein 4 (*FABP4*) at mRNA and protein levels. Pharmacological intervention showed that the *PPARγ* antagonist GW9662 suppressed the pro-lipogenic action of *FABP3* overexpression, while the *PPARα* agonist GW7647 reinstated lipid accumulation in *FABP3*-silenced cells, indicating a reliance on PPAR-pathway activity. *FABP3* therefore drives lipid deposition in adipocytes and hepatocytes via PPAR signaling, offering a framework for how the liver and abdominal adipose tissue jointly govern abdominal fat deposition in broilers.

## 1. Introduction

Modern commercial broilers are the product of many generations of selective breeding aimed at fast growth and better production performance, yet such intense selection often brings greater fat deposition in its wake [1]. Excessive abdominal fat lowers feed efficiency and pushes up production costs [2], and it also impairs reproductive performance [3]. Understanding how abdominal fat is deposited in broilers is therefore a prerequisite for keeping excess fat accumulation in check.

Over the past decade, RNA sequencing (RNA-Seq) and microarray profiling have repeatedly been used to pin down the genes and pathways that control abdominal fat deposition in broilers. Microarray work identified 230 significantly differentially expressed genes (DEGs) that separate fat from lean broilers and assigned them to lipid and energy metabolism pathways [4]. An RNA-Seq comparison of the same contrast returned 286 significant DEGs, among which genes of lipid synthesis were upregulated, while those driving triglyceride hydrolysis were downregulated [5]. Profiling abdominal fat from broilers at 3, 4, 5 and 6 weeks of age by gene microarray likewise uncovered DEGs that distinguish fat from lean birds at each of the four growth stages [6]. Most of these studies examined a single adipose depot, even though abdominal fat deposition is a complex process governed by crosstalk among several tissues, the liver being the principal site of lipid synthesis and abdominal adipose tissue the principal site of storage [7]. How the two tissues coordinate this process has attracted little attention. Because datasets differ, the key genes reported by different groups are often inconsistent, which is why a cross-tissue, multi-dataset integration strategy is valuable for exposing conserved regulators. Meta-analysis integrates multiple independent datasets to effectively improve statistical power and identify more robust conclusions [8]. For example, a meta-analysis of 28 studies clarified the immunosuppressive effects of heat stress [9], whereas another study combining five transcriptomic datasets identified key genes underlying woody breast syndrome [10]. These findings indicate that meta-analysis of multiple datasets is a powerful strategy to identify key genes and reveal conserved molecular mechanisms.

Fatty acid-binding proteins (FABPs) form a family of lipid-binding proteins with characteristic tissue-specific expression patterns whose principal job is to bind and transport hydrophobic fatty acids [11]. One family member, fatty acid-binding protein 3 (*FABP3*), plays an important part in lipid metabolism in adipose and liver tissue alike. Overexpressing *FABP3* in mouse preadipocytes markedly accelerates adipocyte differentiation and raises adipogenic genes, such as glycerol-3-phosphate dehydrogenase and peroxisome proliferator-activated receptor gamma (*PPARγ*) [12]. RNA-Seq analysis has shown *FABP3* to be significantly upregulated in fat relative to lean broilers [13]. The signaling cascades downstream of *FABP3* remain undefined, and what *FABP3* knockdown does during adipogenesis has never been examined in poultry.

The present work therefore set out to integrate three abdominal fat transcriptomic series (GSE42980, GSE49121, GSE104039) with the liver series GSE104038 in one meta-analysis, so as to identify meta-differentially expressed genes (meta-DEGs) linked to abdominal fat deposition across hepatic and adipose tissue. Gene Ontology (GO), Kyoto Encyclopedia of Genes and Genomes (KEGG), gene set enrichment analysis (GSEA) and protein–protein interaction (PPI) network analyses were then applied to the meta-DEGs to screen for key lipid metabolism-related genes. The specific aims of this study were to identify conserved lipid-metabolism genes shared by hepatic and adipose transcriptomes of broilers differing in abdominal fatness, and to determine, in primary chicken preadipocytes and hepatocytes, whether the prioritized candidate *FABP3* regulates cell proliferation, adipogenic differentiation and lipid deposition, and whether these effects depend on peroxisome proliferator-activated receptor (PPAR) signaling pathway activity. By pairing multi-tissue omics with molecular validation, this work lays a foundation for clarifying how abdominal fat deposition is coordinated between hepatic and adipose tissues in broilers.

## 2. Results

### 2.1. Differential Expression Analyses

**Figure 1:**
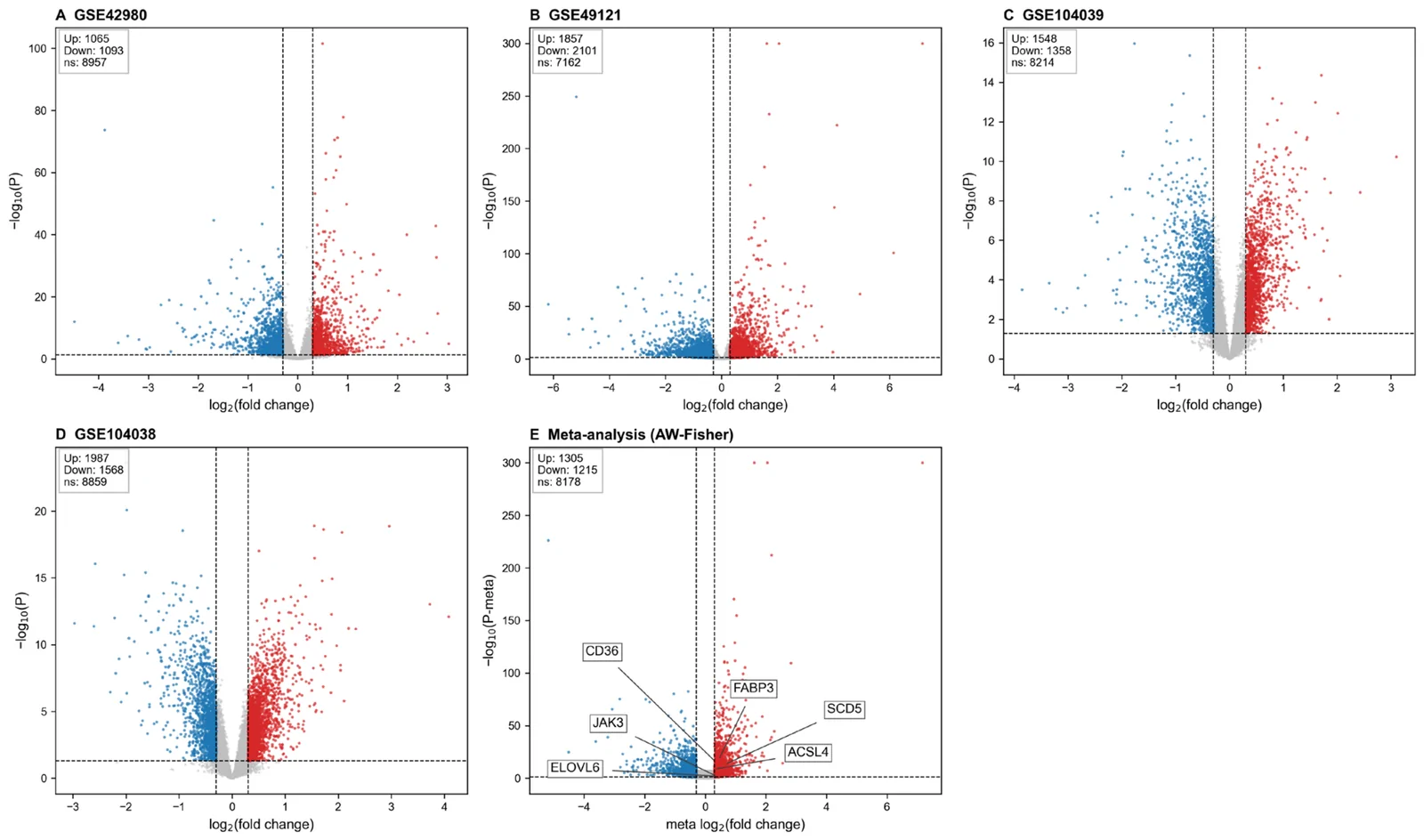
Volcano plots for the individual datasets and for the meta-analysis. (**A**–**D**): volcano plots for GSE42980, GSE49121, GSE104039 and GSE104038, respectively. (**E**): volcano plot of the meta-differentially expressed genes(meta-DEGs) returned by meta-analysis of the four datasets. Panels (**A**–**D**) present each dataset analysed on its own: red dots mark genes with log_2_ fold change (FC) ≥ 0.3 and *p* < 0.05, blue dots mark genes with log_2_FC ≤ −0.3 and *p* < 0.05, and gray dots denote genes that are not significantly differentially expressed (|log2FC| < 0.3 or *p* ≥ 0.05). In panel (**E**), significantly upregulated genes (log_2_FC ≥ 0.3 and *p*-Meta < 0.05) are red, significantly downregulated genes (log_2_FC ≤ −0.3 and *p*-Meta < 0.05) are blue, and gray dots denote genes that are not significantly differentially expressed or that fail the direction-consistency test (|log_2_FC| < 0.3 or *p*-Meta ≥ 0.05 or Direction_Consistent = False). Dashed horizontal lines represent the significance thresholds (*p* = 0.05 for (**A**–**D**); *p*-Meta = 0.05 for (**E**)); dashed vertical lines represent the fold-change thresholds (log_2_FC = ±0.3).

Differential expression was first assessed separately in each dataset, with DESeq2 applied to RNA-Seq datasets and limma to microarray datasets, applying uniform per-dataset criteria of *p* < 0.05 and |log2fold change (FC)| ≥ 0.3. In GSE42980, 2158 DEGs were identified between the fat and lean groups (Figure 1A); in GSE49121, 3958 DEGs were identified (Figure 1B); in GSE104039, 2906 DEGs were identified (Figure 1C); and in GSE104038, 3555 DEGs were identified (Figure 1D). To identify candidate genes, we performed a meta-analysis on the 10,698 genes shared across the four datasets, which comprised 66 fat and 65 lean transcriptomic samples, including 85 abdominal fat and 46 liver samples. There were 2520 significant meta-DEGs identified between the fat-line/ high-fiber and high-fat diet (HF) and lean-line/ standard high-starch (LF) groups (Supporting Information, Table S1). Within the meta-DEGs 1305 genes had higher expression in fat-line/HF groups relative to lean-line/LF groups; *FABP3*, stearoyl-CoA desaturase (*SCD*), proline-rich membrane anchor 1 (*PRIMA1*), and insulin-like growth factor-binding protein 2 (*IGFBP2*) were representative. A further 1215 genes showed lower expression, including enoyl-CoA hydratase and 3-hydroxyacyl CoA dehydrogenase (*EHHADH*), extracellular fatty acid-binding protein (*EXFABP*), and aconitate decarboxylase 1 (*ACOD1*) (Figure 1E, Supporting Information, Table S1). Of the 2520 meta-DEGs, 2512 (99.7%) also passed Benjamini–Hochberg false discovery rate (FDR) < 0.05, indicating that the meta-DEG list was robust to FDR correction.

### 2.2. Kyoto Encyclopedia of Genes and Genomes (KEGG), Gene Ontology (GO) and Protein–Protein Interaction (PPI) Analyses of the Meta-Differentially Expressed Genes(Meta-DEGs)

**Figure 2:**
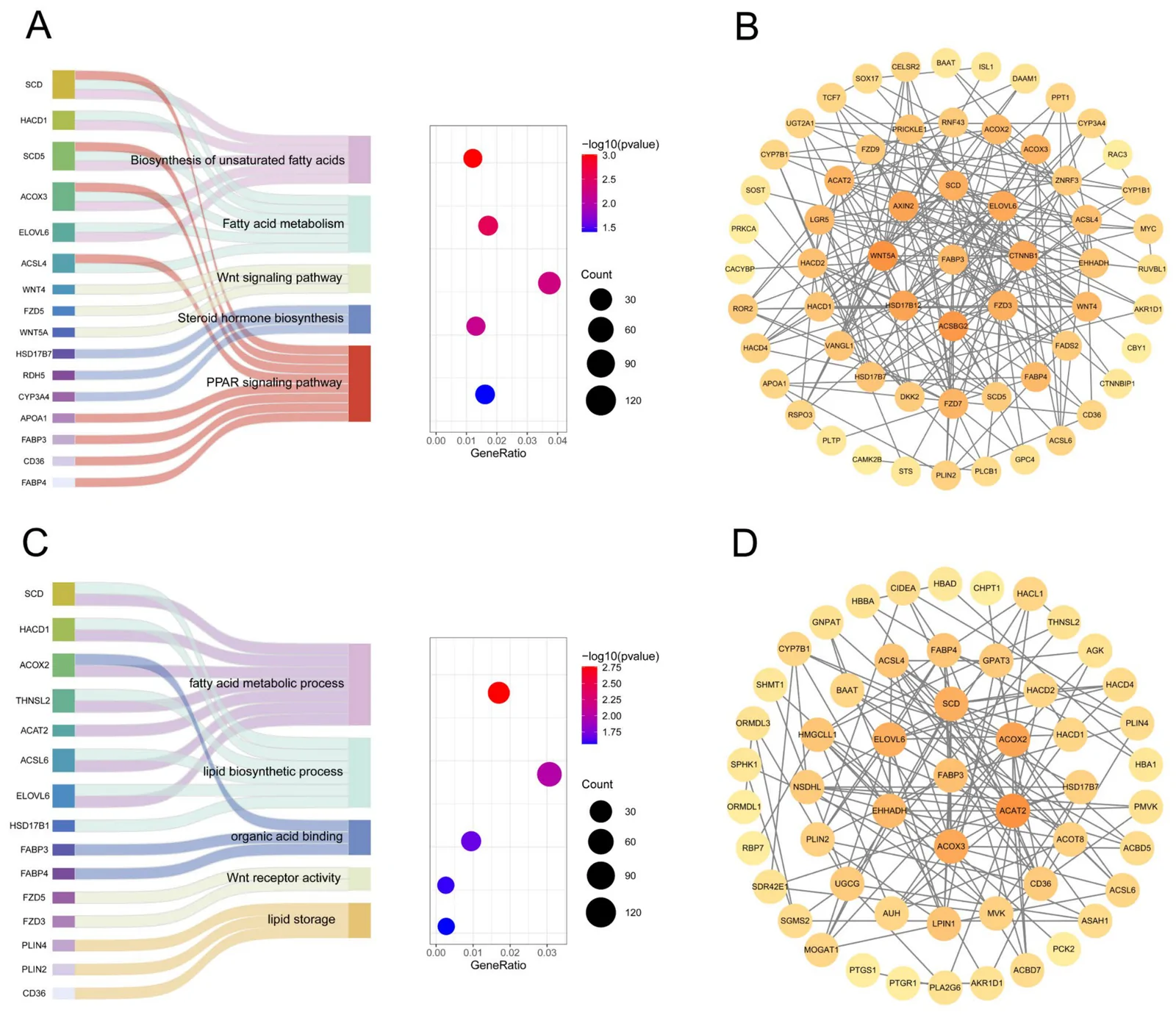
Kyoto Encyclopedia of Genes and Genomes (KEGG) and Gene Ontology (GO) enrichment with the associated meta-DEG protein–protein interaction (PPI) network. (**A**): KEGG enrichment. The left-hand Sankey diagram connects genes with the pathways they belong to. These pathways are then arranged along the vertical axis of the bubble diagram on the right, whose horizontal axis reports how strongly the meta-DEGs are enriched in each pathway. Bubble color carries the enrichment significance, with deeper red meaning greater significance. (**B**): PPI network construction based on KEGG-enriched pathways. Nodes with darker colors indicate more significant genes. (**C**): GO enrichment, displayed in the same two-panel format: genes are linked to their pathways by the Sankey diagram on the left, and the bubble diagram on the right again sets out those pathways vertically against the enrichment level horizontally, with color encoding significance as before. (**D**): PPI network construction based on GO-enriched pathways. Nodes with darker colors indicate more significant genes.

According to the KEGG database, 32 pathways reached significance among the meta-DEGs (Supporting Information, Table S2), and five of them are related to lipid metabolism: the PPAR signaling pathway, fatty acid metabolism, biosynthesis of unsaturated fatty acids, the Wnt signaling pathway and steroid hormone biosynthesis. Within these five pathways, *SCD*, *FABP3*, stearoyl-CoA desaturase 5 (*SCD5*), Wnt family member 5A (*WNT5A*) and *EHHADH* were identified (Figure 2A). A PPI network of the meta-DEGs contained in these five lipid metabolism pathways was then constructed (Figure 2B).

In the GO analysis, 349 terms were significantly enriched among the meta-DEGs, comprising 254 Biological Process (BP), 35 Cellular Component (CC) and 60 Molecular Function (MF) terms (Supporting Information, Table S3). Within the BP terms, lipid biosynthetic process and fatty acid metabolic process were enriched. The CC terms included lysosome and vacuole, and organic acid binding together with extracellular matrix binding stood out among the MF terms. Of the 349 GO terms, 18 were related to lipid metabolism, and the genes they contained included acyl-CoA oxidase 2 (*ACOX2*), *FABP3*, *SCD*, 3-hydroxyacyl-CoA dehydratase 1 (*HACD1*), lipin 1 (*LPIN1*), solute carrier family 38 member 1 (*SLC38A1*) and fatty acid-binding protein 4 (*FABP4*). The five-lipid metabolism-related GO terms were displayed as a Sankey bubble diagram (Figure 2C), and a PPI network was built from the meta-DEGs contained in those five terms (Figure 2D).

### 2.3. Gene Set Enrichment Analysis (GSEA) and PPI of the Meta-DEGs

**Figure 3:**
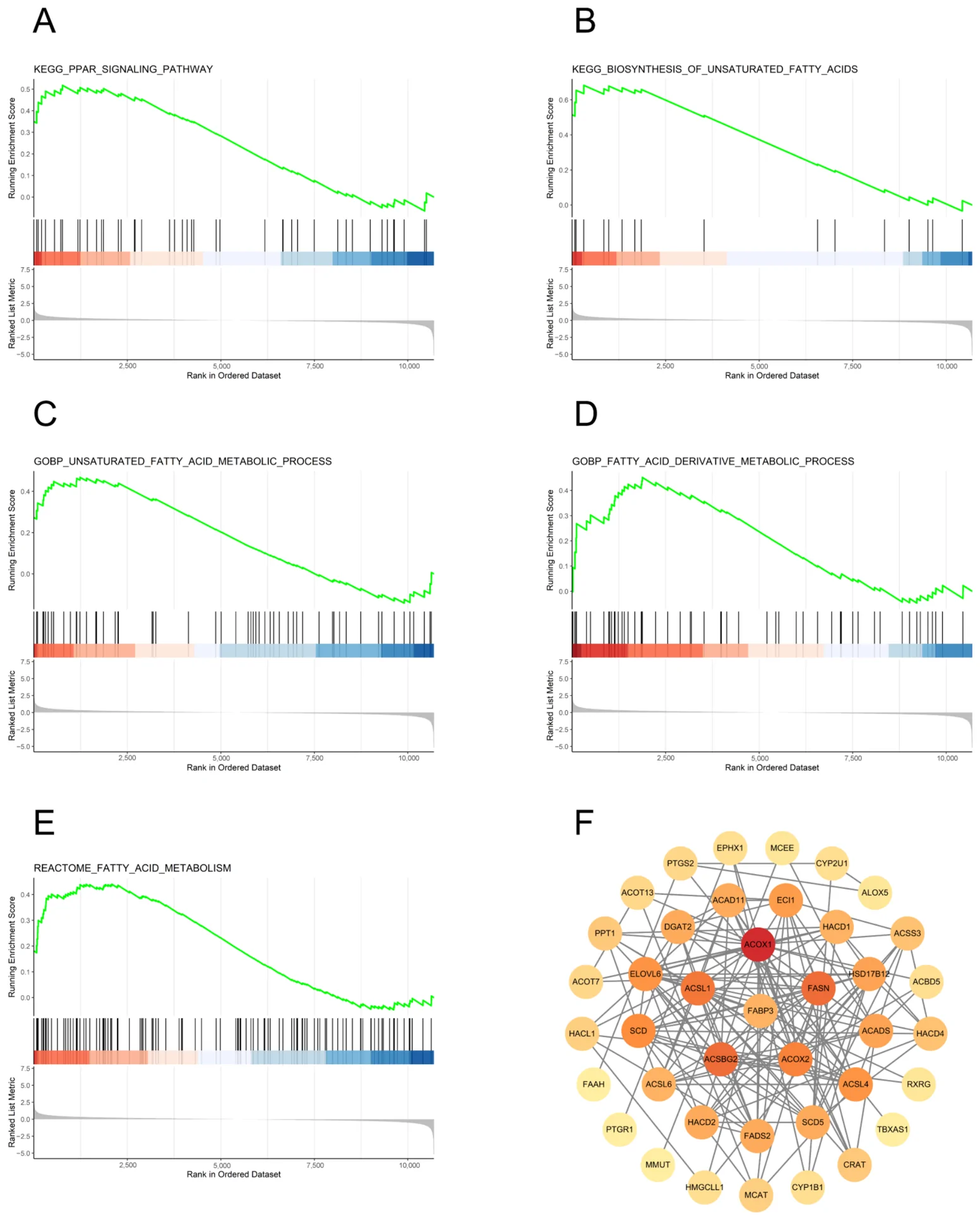
Enrichment of the five foremost lipid metabolism gene sets and the PPI network derived from them. (**A**–**E**): gene set enrichment analysis (GSEA) plots for the five gene sets most strongly upregulated in relation to lipid metabolism. Genes are colored in red for upregulation and blue for downregulation. (**F**): PPI network built from the GSEA-enriched pathways, showing how significant the participating lipid-metabolism genes are. Darker nodes mark greater significance.

GSEA returned 372 significantly enriched gene sets, 19 of which are associated with lipid metabolism (Supporting Information, Table S4). The five gene sets with the lowest *p*-values—‘fatty acid metabolism’, ‘PPAR signaling pathway’, ‘biosynthesis of unsaturated fatty acids’, ‘unsaturated fatty acid metabolic process’ and ‘fatty acid derivative metabolic process’—were visualized (Figure 3A–E), and genes of interest, such as acyl-CoA synthetase long-chain family member 4 (*ACSL4*) and *FABP3* were identified. A PPI network built from the meta-DEGs of these five gene sets is shown in Figure 3F.

### 2.4. Consistency, Heterogeneity and Sensitivity of Fatty Acid-Binding Protein 3 (FABP3) Expression Across Datasets

To describe how *FABP3* behaves across the four datasets, its log2FC was computed for every individual comparison (Supporting Information, Table S6). Upregulation in the fat-line/HF groups was seen consistently: log2FC = +0.60 (GSE42980), +0.46 (GSE49121), +0.33 (GSE104039) and +0.44 (GSE104038). The corresponding back-calculated SEs were 0.0846, 0.0927, 0.0827 and 0.1879. Heterogeneity between datasets was moderate and did not reach significance (Cochran’s Q = 5.43, df = 3, *p* = 0.14; I^2^ = 44.76%; τ^2^ = 0.008) (Supporting Information, Table S7), indicating that the differences among datasets, while present, were too small to be statistically meaningful and left the consistency of *FABP3* upregulation intact. Pooling under a fixed-effect model afforded log2FC = +0.46 (95% CI: 0.37–0.55; *p* = 1.2 × 10^−21^); under a random-effects (DerSimonian–Laird) model, the pooled log2FC was again +0.46 (95% CI: 0.33–0.59; *p* = 1.3 × 10^−11^), confirming that *FABP3* is robustly and significantly upregulated. In the Supporting Information, Figure S1 presents the forest plot of *FABP3* across the four datasets. In the leave-one-out sensitivity analysis, dropping any single dataset left pooled log2FC values between +0.39 and +0.53, and restricting the analysis to adipose tissue (that is, omitting the hepatic series GSE104038) yielded a pooled log2FC of +0.46 (95% CI 0.30–0.62) (Supporting Information, Table S8).

These results demonstrate that no single dataset or tissue drove the pooled estimate. To prioritize among the recurrently enriched lipid-metabolism genes, we applied three additional criteria: presence in all four datasets with an adaptive weight of 1, nominal significance (*p* < 0.05) in every dataset, and consistent direction of change across datasets. Eleven meta-DEGs were recurrently enriched across KEGG, GO and GSEA, including *FABP3*, *SCD*, *ACSL4*, *ELOVL6*, *ACOX2*, *HACD1* and *HACD2*. Of these, only *FABP3*, *HACD2* and *ACSL4* satisfied all three criteria. Among the three shortlisted genes, *FABP3* showed the strongest combined evidence (AW-Fisher *p* = 3.2 × 10^−19^; *HACD2*, 5.5 × 10^−19^; *ACSL4*, 1.5 × 10^−9^), occupied a central fatty-acid transport/chaperone node upstream of PPAR signaling, and lacked prior functional characterization in poultry. These criteria are detailed in the Supporting Information, Table S6, and explain why *FABP3* was selected over *SCD* and other candidates.

### 2.5. FABP3 Stimulates Proliferation of Primary Chicken Preadipocytes

**Figure 4:**
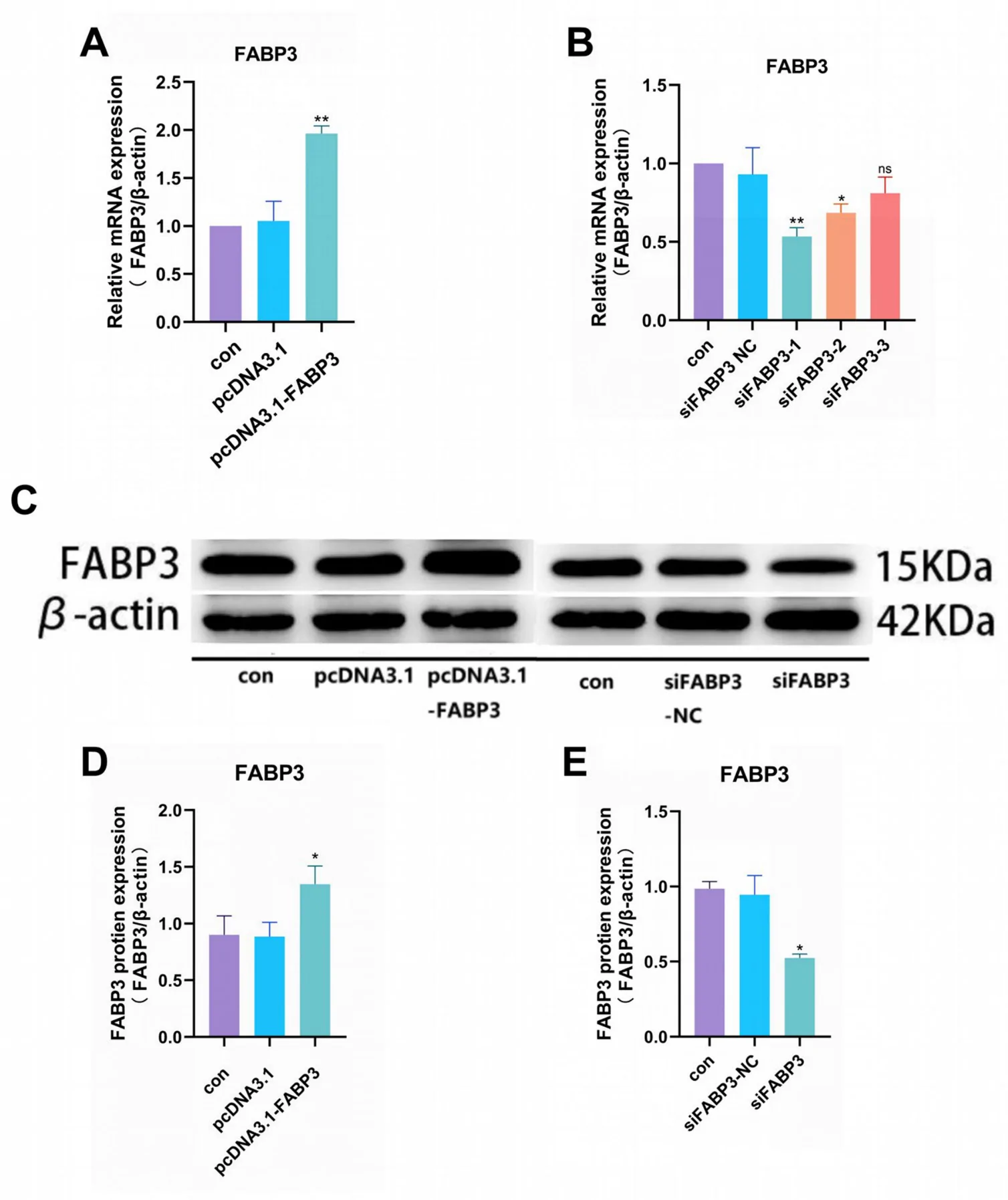
*FABP3* transfection efficiency in primary chicken preadipocytes. (**A**): *FABP3* mRNA quantified in *FABP3*-overexpressing cells, expressed relative to *β-actin*. (**B**): *FABP3* mRNA quantified in *FABP3*-silenced cells, expressed relative to *β-actin*. (**C**): detection of FABP3 protein by Western blot (WB). (**D**): FABP3 protein quantified in *FABP3*-overexpressing cells, normalized to the β-actin (42 kDa) loading control. (**E**): FABP3 protein quantified in *FABP3*-silenced cells, likewise normalized to *β-actin*. Three replicates were run per experiment (*n* = 3). Asterisks: * *p* < 0.05, ** *p* < 0.01; ns, not significant.

qPCR showed that cells receiving pcDNA3.1-*FABP3* contained markedly more *FABP3* transcript than the control (con) or empty overexpression vector (pcDNA3.1) cells (*p* < 0.01; Figure 4A). Conversely, *FABP3* mRNA fell below con and si*FABP3*-NC levels in each of the three knockdown groups, the reduction reaching significance for si*FABP3*-1 (*p* < 0.01) and si*FABP3*-2 (*p* < 0.05) but not for si*FABP3*-3 (ns; Figure 4B).

Western blotting reproduced this pattern at the protein level: *FABP3* abundance rose in pcDNA3.1-*FABP3*-transfected cells relative to con and pcDNA3.1 (*p* < 0.05; Figure 4C,D), and fell in si*FABP3* cells compared with con and si*FABP3*-NC (*p* < 0.05; Figure 4C,E). The si*FABP3*-1 exhibited the highest knockdown efficiency among the three screened siRNA sequences, and was therefore used for all subsequent loss-of-function experiments, including the pathway-intervention assays. These results confirmed the successful establishment of si*FABP3* and pcDNA3.1-*FABP3* models, providing a foundation for subsequent experiments.

**Figure 5:**
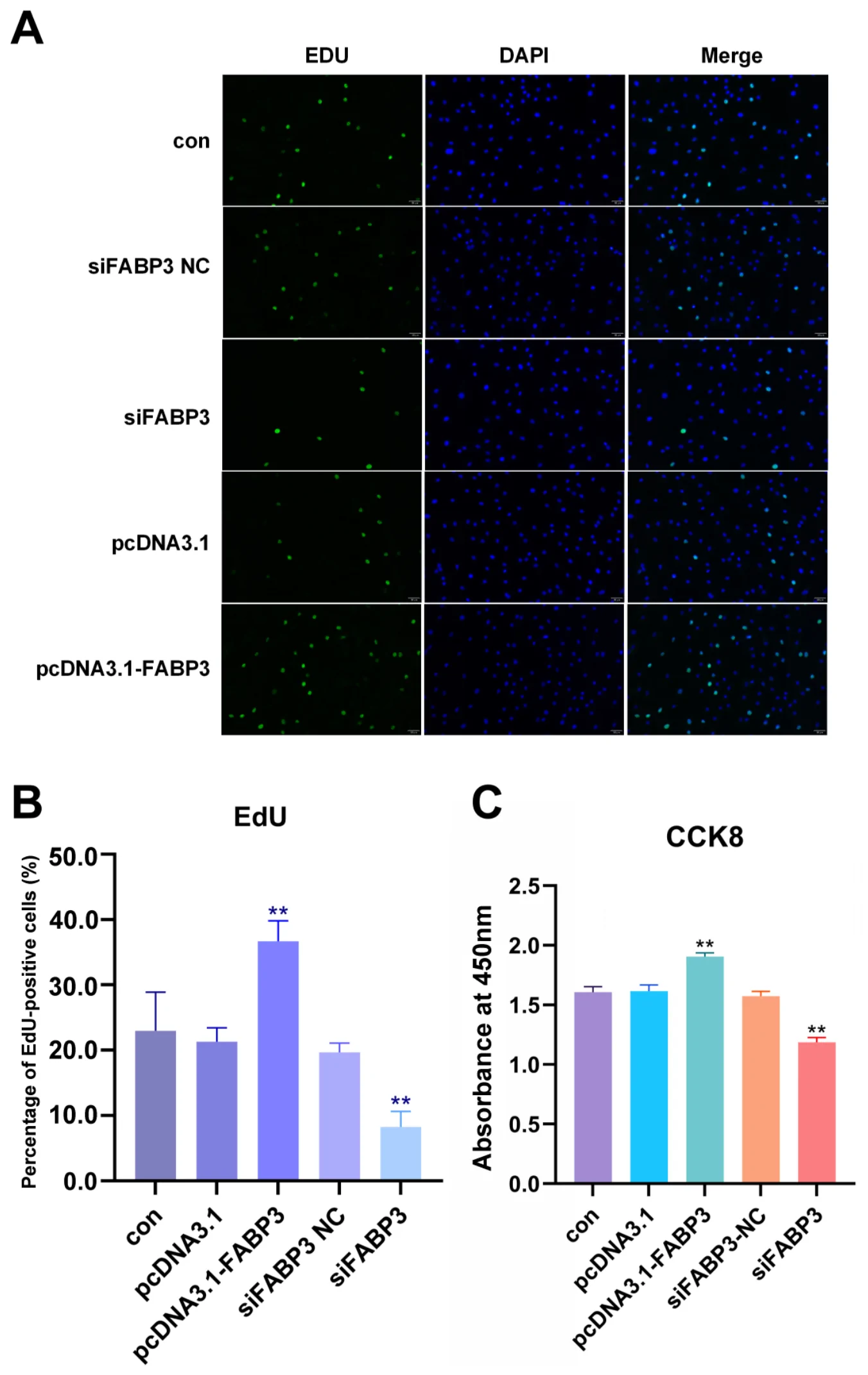
*FABP3* and the proliferation of primary chicken preadipocytes. (**A**): EdU/DAPI immunofluorescence staining of primary chicken preadipocytes, with proliferating nuclei clearly labeled (scale bar = 50 μm). (**B**): percentage of EdU-positive cells (%). (**C**): proliferative activity of primary chicken preadipocytes at 72 h measured by the CCK-8 assay and expressed as absorbance (OD450). The dataset rests on three biological replicates (*n* = 3). CCK-8 readings were averaged across the six technical wells of each group within a replicate before statistical analysis. EdU positivity came from three randomly chosen fields per coverslip. Significance: ** *p* < 0.01.

EdU labeling identified a larger proportion of proliferating cells after *FABP3* overexpression than in either the con or the pcDNA3.1 group, whereas si*FABP3* reduced that proportion below the levels seen in con and si*FABP3*-NC cells (*p* < 0.01 for both comparisons; Figure 5A,B). Consistent with this, the CCK-8 assay recorded higher viability at 72 h in pcDNA3.1-*FABP3* cells (blank-corrected absorbance above 1.8) and lower viability in si*FABP3* cells, each relative to its own controls (*p* < 0.01 in both cases; Figure 5C).

### 2.6. FABP3 Promotes Adipogenesis in Chicken Adipocytes

**Figure 6:**
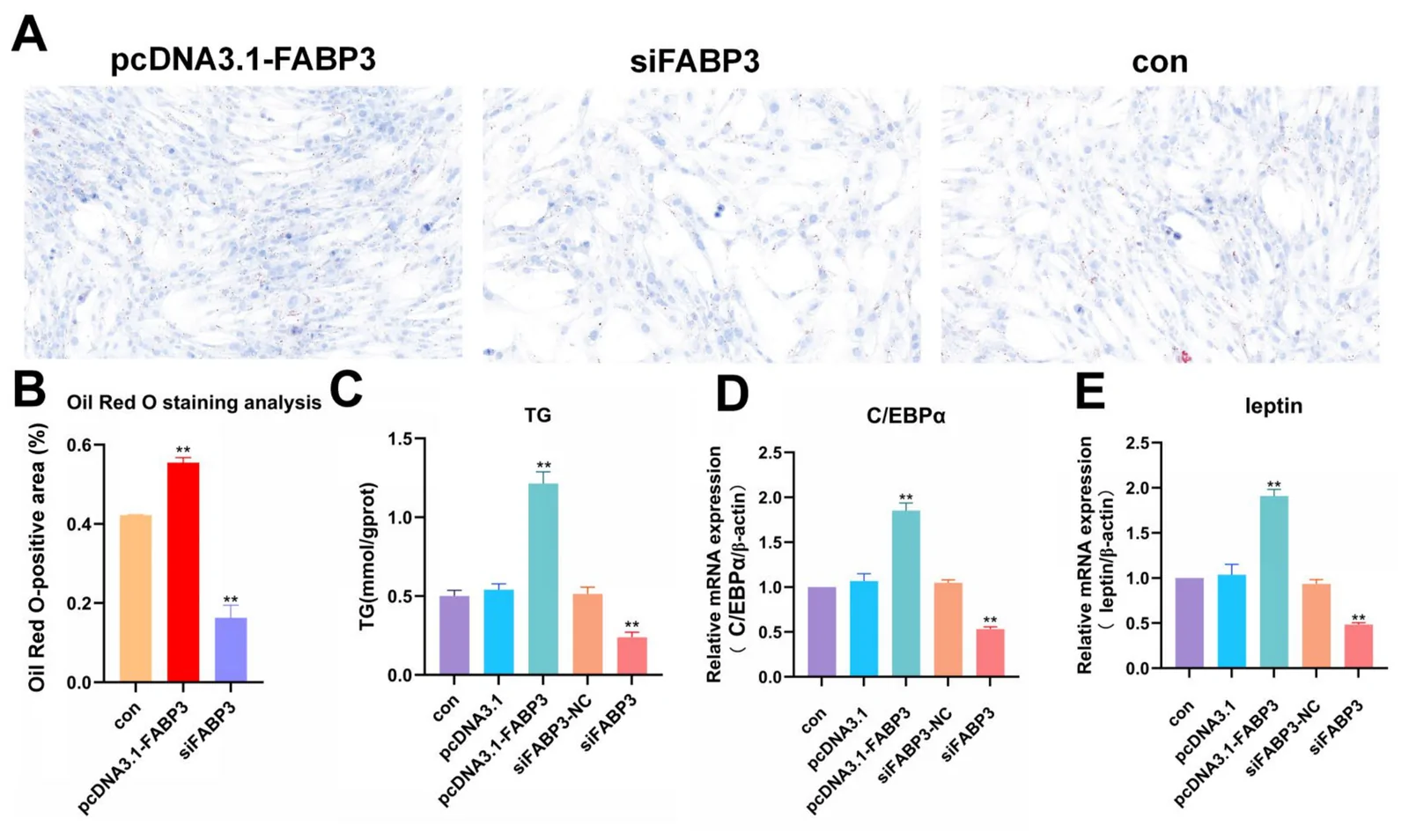
Effect of *FABP3* on lipid accumulation in primary chicken adipocytes. (**A**): Oil Red O-stained images showing intracellular lipid droplet accumulation. (**B**): quantification of Oil Red O staining (% Oil Red O-positive area). (**C**): measurement of TG content. (**D**): abundance of the adipogenic transcription factor *C/EBPα* mRNA, expressed relative to *β-actin*. (**E**): leptin mRNA abundance, likewise normalized to *β-actin*. Three biological replicates underlie the data (*n* = 3). Probability values are ** *p* < 0.01.

At 96 h of differentiation, Oil Red O staining covered a significantly greater stained area in pcDNA3.1-*FABP3* cells than in con cells, while si*FABP3* cells stained far less intensely than con cells at the same time point (*p* < 0.01 for both; Figure 6A,B). Intracellular triglyceride (TG) content followed the same trajectory, rising above con and pcDNA3.1 levels upon *FABP3* overexpression (*p* < 0.01; Figure 6C) and falling below con and si*FABP3*-NC levels after silencing (*p* < 0.01; Figure 6C). Transcripts of the adipogenic markers *C/EBPα* and *leptin* were likewise elevated by overexpression and depressed by silencing (*p* < 0.01 throughout; Figure 6D,E).

### 2.7. FABP3 Promotes Lipid Accumulation via the Peroxisome Proliferator-Activated Receptor (PPAR) Signaling in Primary Chicken Adipocytes

**Figure 7:**
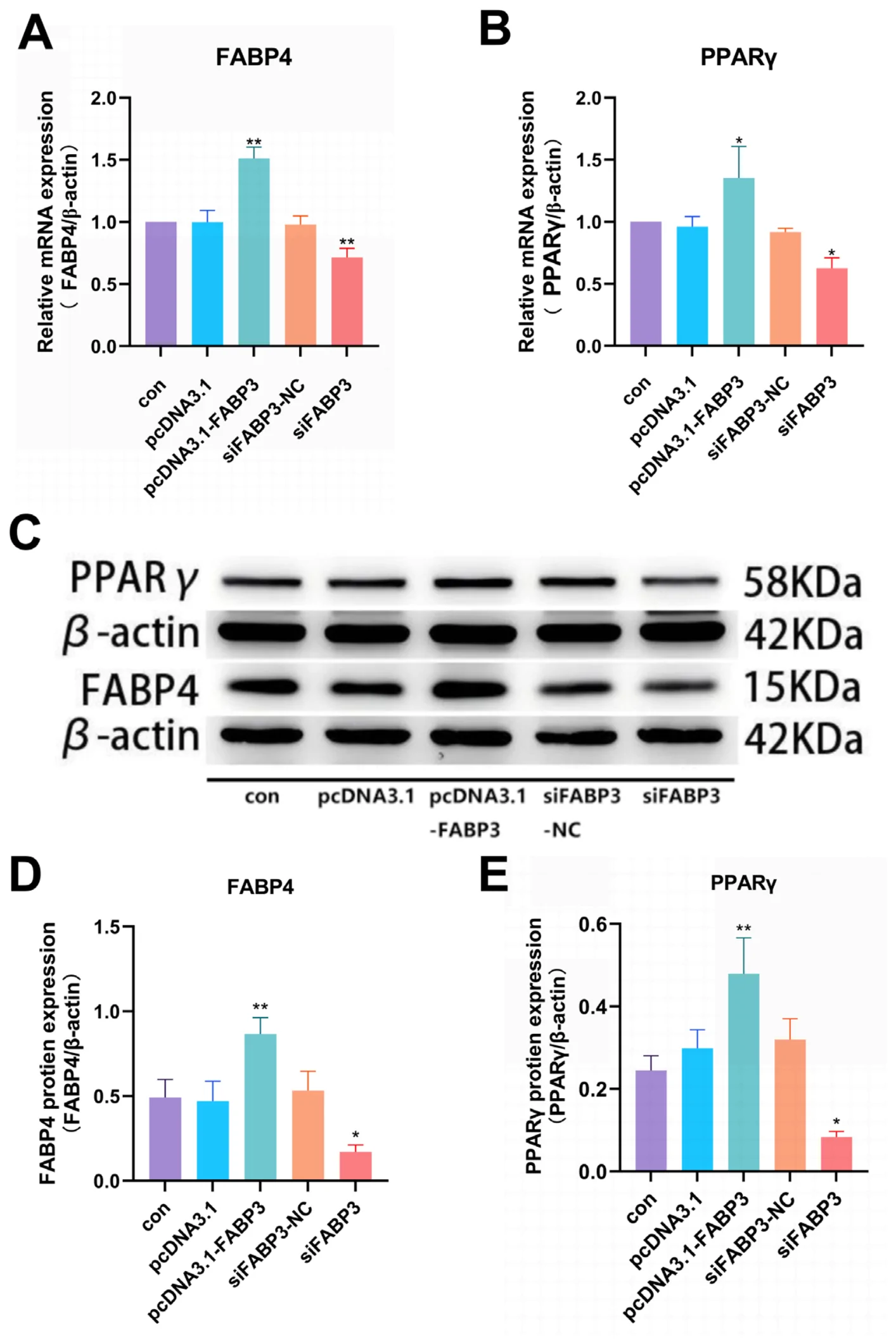
*FABP4* and *PPARγ* expression in primary chicken adipocytes as affected by *FABP3*. (**A**): *FABP4* mRNA abundance relative to *β-actin*. (**B**): *PPARγ* mRNA abundance relative to *β-actin*. (**C**): WB detection of PPARγ and FABP4 protein. (**D**): relative FABP4 protein level; the β-actin (42 kDa) band provided the loading control. (**E**): relative PPARγ protein level, with the same loading control. The data come from three independent biological replicates (*n* = 3). Significance levels are * *p* < 0.05; ** *p* < 0.01.

*FABP4* and *PPARγ* transcripts both rose after *FABP3* overexpression, the former by *p* < 0.01 and the latter by *p* < 0.05 (Figure 7A,B); silencing reversed this, with *FABP4* (*p* < 0.01) and *PPARγ* (*p* < 0.05) falling below their control levels (Figure 7A,B). Western blots showed the same direction of change: PPARγ and FABP4 protein were both elevated by pcDNA3.1-*FABP3* (*p* < 0.01 each; Figure 7C–E) and both suppressed by si*FABP3* (*p* < 0.05 each; Figure 7C–E).

**Figure 8:**
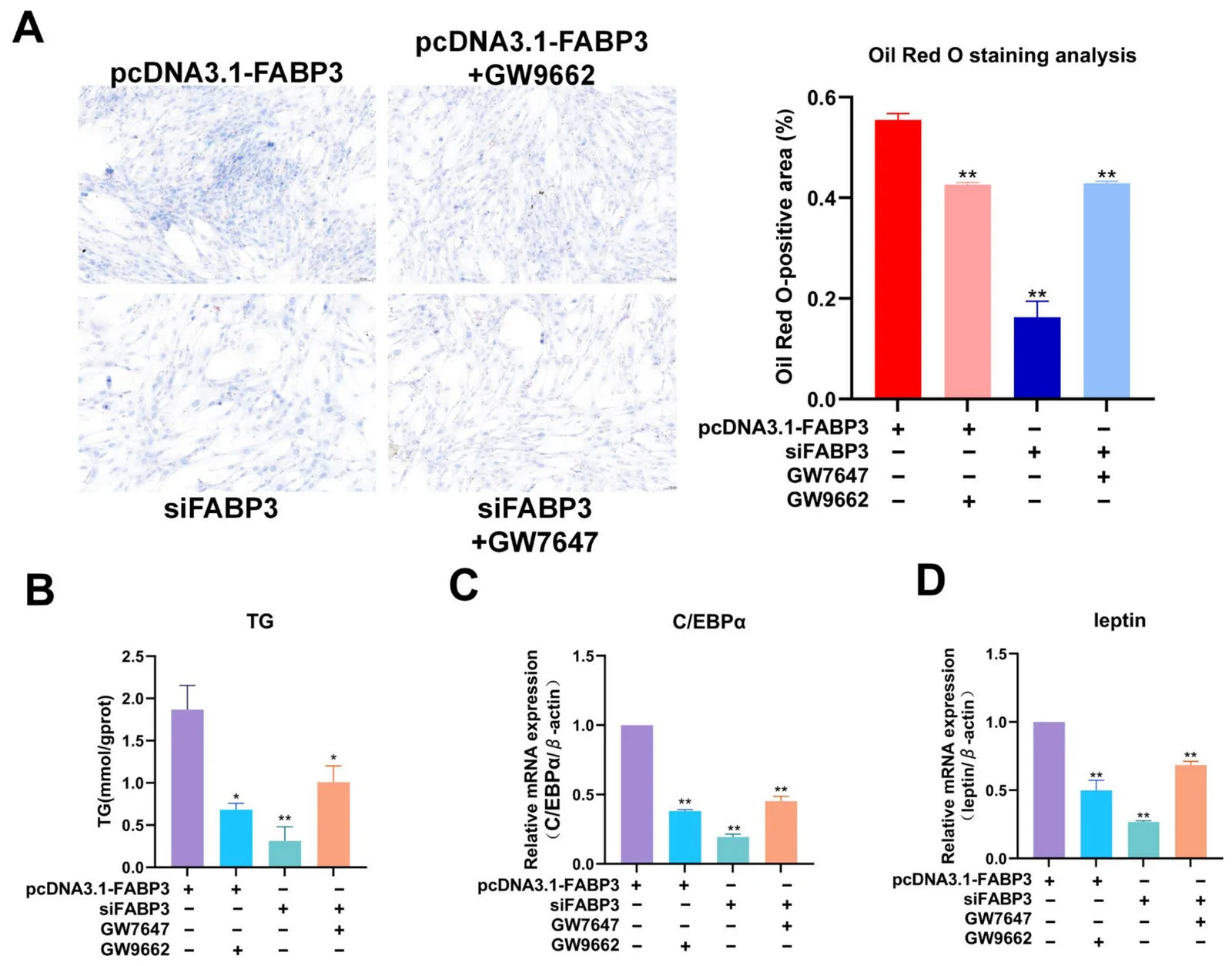
Effect of *FABP3* on PPAR signaling pathway intervention-mediated lipid accumulation in primary chicken adipocytes. (**A**): Oil Red O-stained images of intracellular lipid droplet accumulation at 96 h together with quantification (% Oil Red O-positive area). (**B**): TG content. (**C**): *C/EBPα* mRNA relative to *β-actin*. (**D**): leptin mRNA relative to *β-actin*. Four treatments are compared: pcDNA3.1-*FABP3*, pcDNA3.1-*FABP3* + *PPARγ* antagonist (GW9662), si*FABP3* and si*FABP3* + *PPARα* agonist (GW7647). Three biological replicates were analysed (*n* = 3). Significance is coded by asterisks: * *p* < 0.05; ** *p* < 0.01.

Adding the *PPARγ* antagonist GW9662 to *FABP3*-overexpressing cultures curtailed the lipid-positive area at 96 h (*p* < 0.01; Figure 8A), whereas the *PPARα* agonist GW7647 enlarged it in *FABP3*-silenced cultures at the same endpoint (*p* < 0.01; Figure 8A). GW9662 likewise lowered *C/EBPα* and *leptin* transcripts (*p* < 0.01) and triglyceride (TG) content (*p* < 0.05) in the overexpression background (Figure 8B–D); conversely, GW7647 raised *C/EBPα* and *leptin* mRNA (*p* < 0.01) and TG content (*p* < 0.05) in the knockdown background (Figure 8B–D).

### 2.8. FABP3 Promotes Proliferation of Primary Chicken Hepatocytes

**Figure 9:**
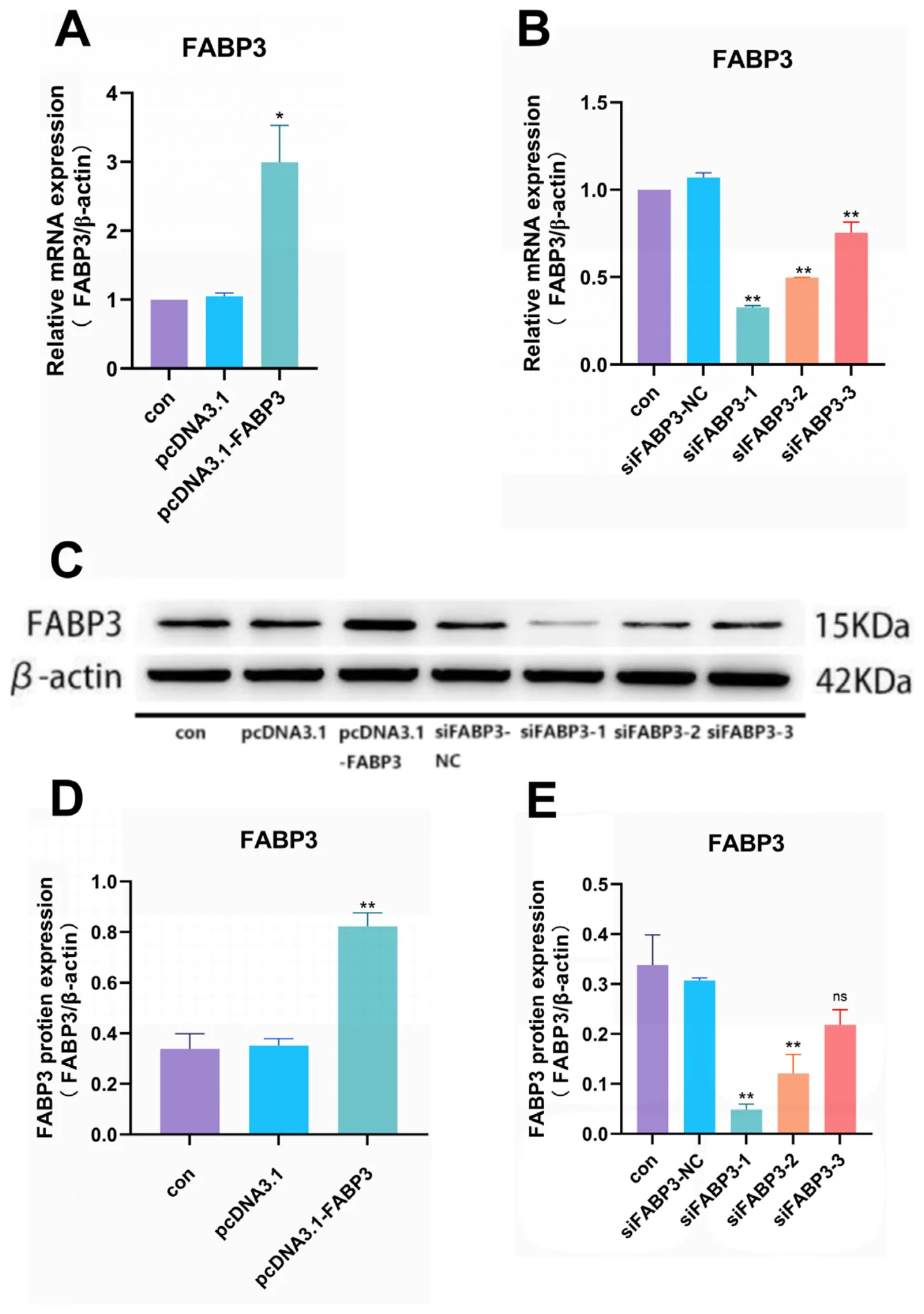
*FABP3* Transfection Efficiency in primary chicken hepatocytes. (**A**): *FABP3* mRNA in the con, pcDNA3.1 and pcDNA3.1-*FABP3* groups, expressed relative to the reference gene *β-actin*. (**B**): *FABP3* mRNA in the con, si*FABP3*NC and si*FABP3* groups, again relative to *β-actin*. (**C**): WB detection of FABP3 protein across the overexpression control, pcDNA3.1, pcDNA3.1-*FABP3*, interference control, si*FABP3*NC and si*FABP3* groups. (**D**): FABP3 protein in the con, pcDNA3.1 and pcDNA3.1-*FABP3* groups, with *β-actin* as reference. (**E**): FABP3 protein in the con, si*FABP3*NC and si*FABP3* groups, *β-actin* again serving as reference. Three replicates per group were used (*n* = 3). Significance is shown as * *p* < 0.05, ** *p* < 0.01; ns, non-significant.

Transfection of hepatocytes with pcDNA3.1-*FABP3* lifted *FABP3* mRNA above the control and pcDNA3.1 levels (*p* < 0.05; Figure 9A), while si*FABP3*-1, si*FABP3*-2 and si*FABP3*-3 each lowered it (*p* < 0.01; Figure 9B). Protein analysis agreed: FABP3 abundance increased after overexpression (*p* < 0.01; Figure 9C,D) and decreased after knockdown, significantly so for si*FABP3*-1 and si*FABP3*-2 (both *p* < 0.01) but not for si*FABP3*-3 (ns; Figure 9C,E). As in preadipocytes, si*FABP3*-1 proved the most effective duplex in primary chicken hepatocytes.

**Figure 10:**
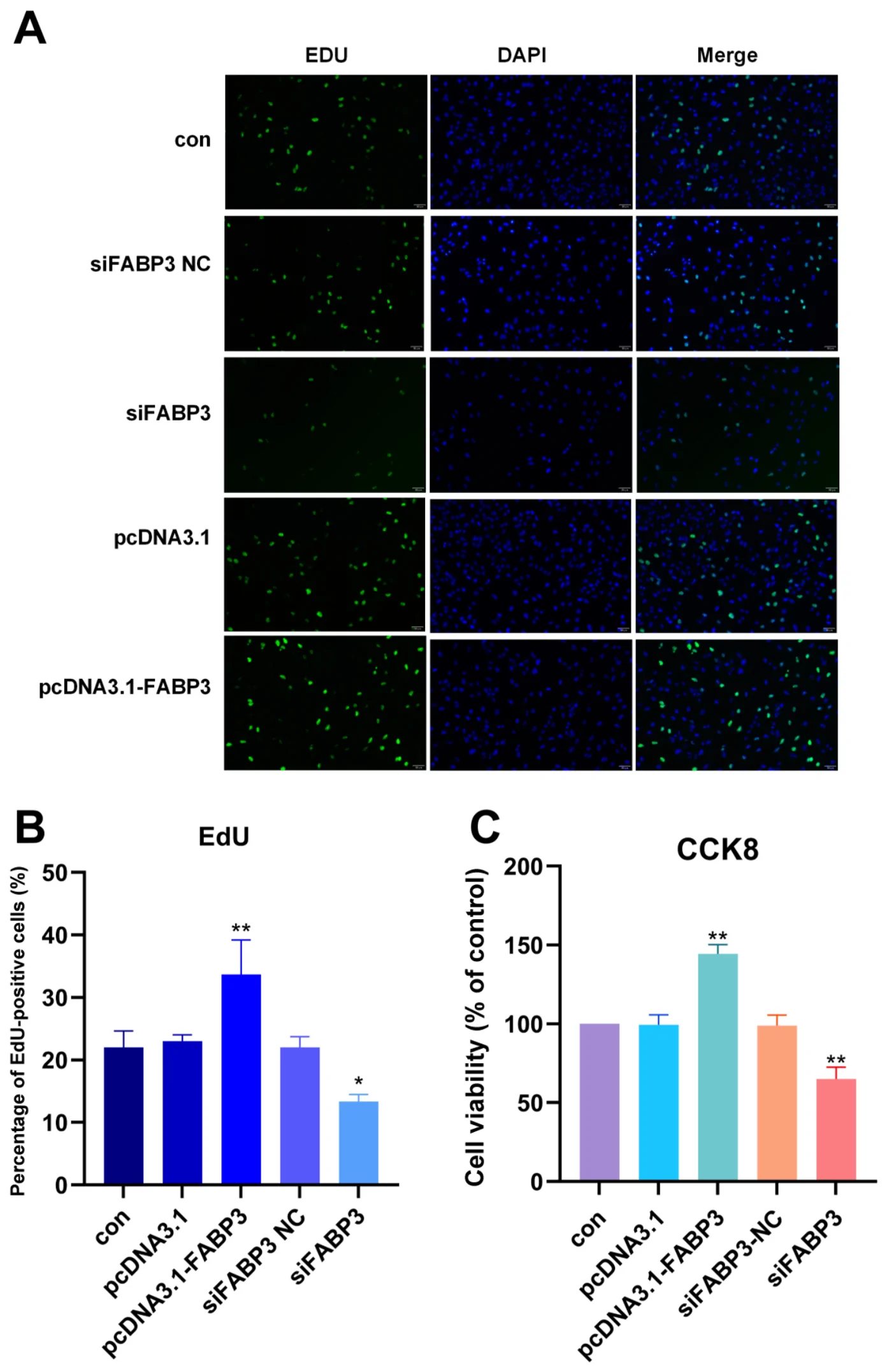
Effects of *FABP3* on the proliferation of chicken primary hepatocytes. (**A**): EdU/DAPI immunofluorescence staining of chicken primary hepatocytes from each group, showing proliferation (scale bar = 50 μm). (**B**): EdU-positive cell percentage (%). (**C**): cell viability of chicken primary hepatocytes in each group at 72 h, measured by CCK-8 and cell viability being expressed as a percentage of the con group. The experiment comprised three biological replicates (*n* = 3). Within each replicate, CCK-8 values were averaged over the six technical wells per group, and EdU counts over three random fields per coverslip, before statistical analysis. Asterisks mark * *p* < 0.05; ** *p* < 0.01.

Hepatocyte proliferation behaved similarly: the fraction of EdU-positive nuclei expanded after *FABP3* overexpression versus con and pcDNA3.1 (*p* < 0.01; Figure 10A,B) and contracted after silencing versus con and si*FABP3*-NC (*p* < 0.05; Figure 10A,B). CCK-8 measurements at 72 h corroborated the proliferative effect, with pcDNA3.1-*FABP3* cells exceeding (*p* < 0.01) and si*FABP3* cells trailing (*p* < 0.01) their respective controls (Figure 10C), together indicating that *FABP3* boosts proliferation and viability in both primary chicken preadipocytes and hepatocytes.

### 2.9. FABP3 Promotes Lipid Deposition in Primary Chicken Hepatocytes

**Figure 11:**
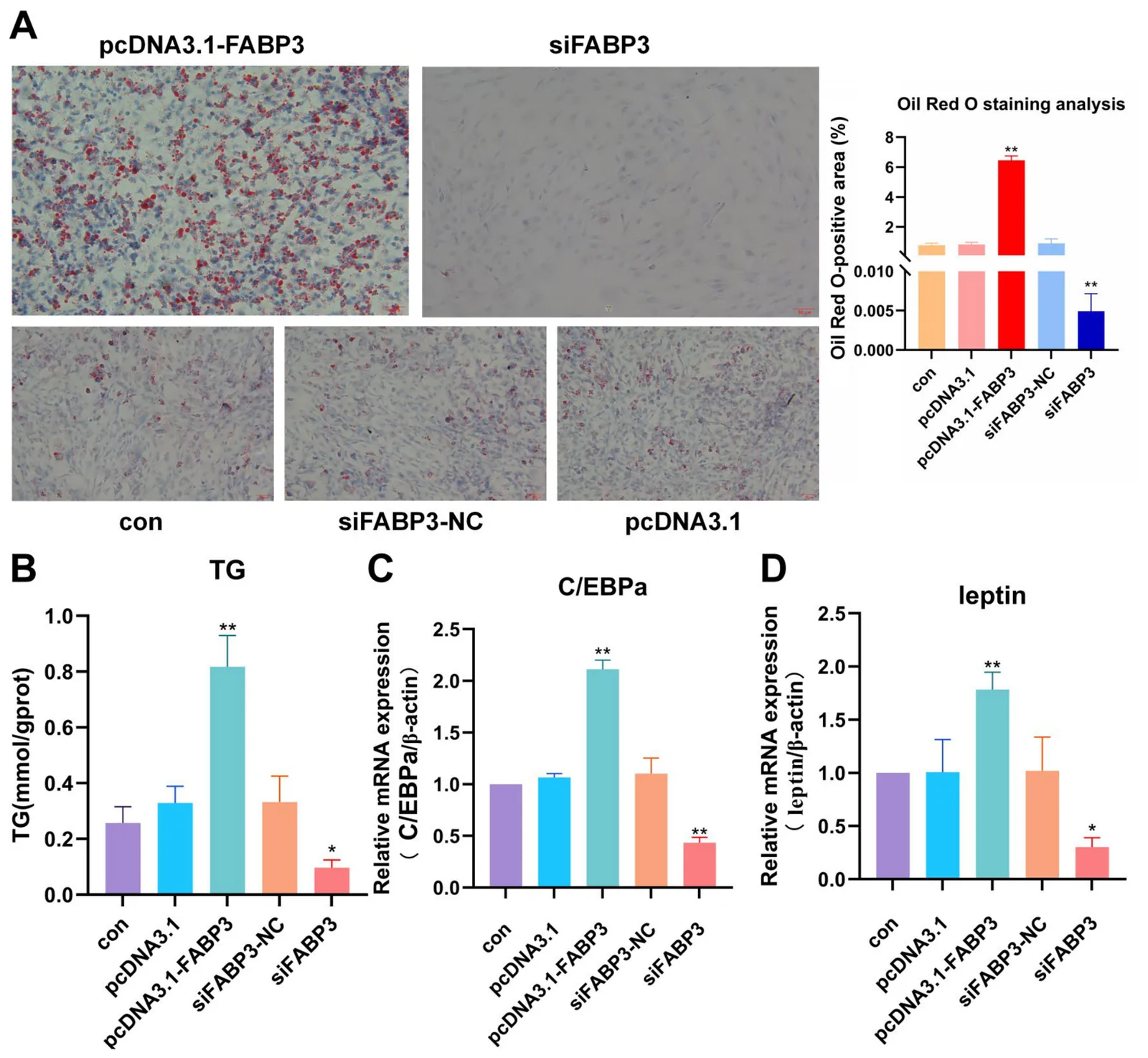
Effect of *FABP3* on lipid deposition in primary chicken hepatocytes. (**A**): representative Oil Red O-stained images with quantification of the percentage of Oil Red O-positive area in each group. (**B**): TG content, the abscissa listing the treatment groups (con, pcDNA3.1, pcDNA3.1-*FABP3*, si*FABP3*-NC, si*FABP3*) and the ordinate providing TG content (mmol/gprot). (**C**): *C/EBPα* mRNA normalized to the reference *β-actin*. (**D**): leptin mRNA normalized to the reference *β-actin*. Each group was tested in three biological replicates (*n* = 3). Asterisks indicate * *p* < 0.05, ** *p* < 0.01.

In differentiating hepatocytes, the Oil Red O-positive area at 96 h was enlarged by *FABP3* overexpression and reduced by silencing, each relative to its corresponding controls (*p* < 0.01 in both cases; Figure 11A). TG accumulation paralleled the staining data, increasing in pcDNA3.1-*FABP3* cells (*p* < 0.01 versus con and pcDNA3.1) and decreasing in si*FABP3* cells (*p* < 0.05 versus con and si*FABP3*-NC; Figure 11B). The mRNA expression levels of key lipogenic genes *C/EBPα* and *leptin* were significantly higher in the pcDNA3.1-*FABP3* than in the con and pcDNA3.1 groups, with both *p* < 0.01 (Figure 11C,D). Conversely, compared with the con and si*FABP3*-NC groups, the si*FABP3* exhibited significantly lower mRNA expression levels of *C/EBPα* and *leptin*, with *p* < 0.01 for *C/EBPα*, and *p* < 0.05 for *leptin* (Figure 11C,D). These results demonstrated that *FABP3* has a consistent facilitatory effect on lipid deposition in hepatocytes and adipocytes.

### 2.10. FABP3 Promotes Lipid Deposition in Primary Chicken Hepatocytes via PPAR Signaling

**Figure 12:**
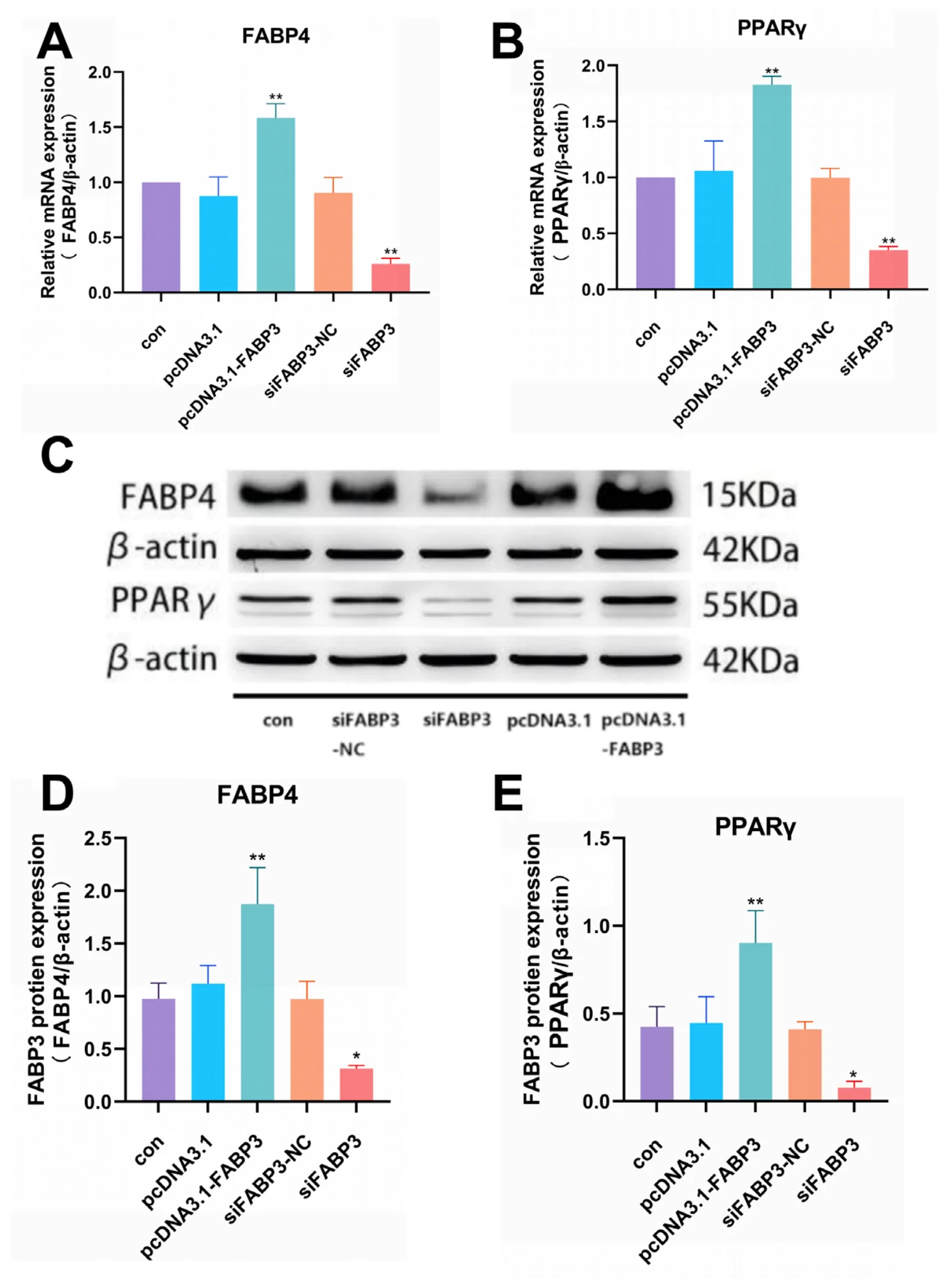
*FABP4* and *PPARγ* expression in hepatocytes as affected by *FABP3*. (**A**): *FABP4* mRNA in chicken primary hepatocytes, expressed relative to the reference *β-actin*. (**B**): *PPARγ* mRNA in chicken primary hepatocytes, likewise relative to *β-actin*. (**C**): WB analysis of FABP4 and PPARγ protein in chicken primary hepatocytes, β-actin (42 kDa) serving as the internal reference. (**D**): relative FABP4 protein in chicken primary hepatocytes, with *β-actin* as reference. (**E**): relative PPARγ protein in chicken primary hepatocytes, again with *β-actin* as reference. Three biological replicates were run (*n* = 3). Asterisks in all panels: * *p* < 0.05, ** *p* < 0.01.

Within hepatocytes, *FABP3* overexpression lifted *FABP4* and *PPARγ* mRNA above con and pcDNA3.1 levels (both *p* < 0.01; Figure 12A,B), and knockdown depressed both transcripts below con and si*FABP3*-NC levels (both *p* < 0.01; Figure 12A,B). At the protein level, pcDNA3.1-*FABP3* increased *PPARγ* and *FABP4* (both *p* < 0.01; Figure 12C–E), while si*FABP3* diminished them (both *p* < 0.05; Figure 12C–E), establishing that *FABP3* drives *PPARγ* and *FABP4* expression in chicken adipocytes and hepatocytes alike.

**Figure 13:**
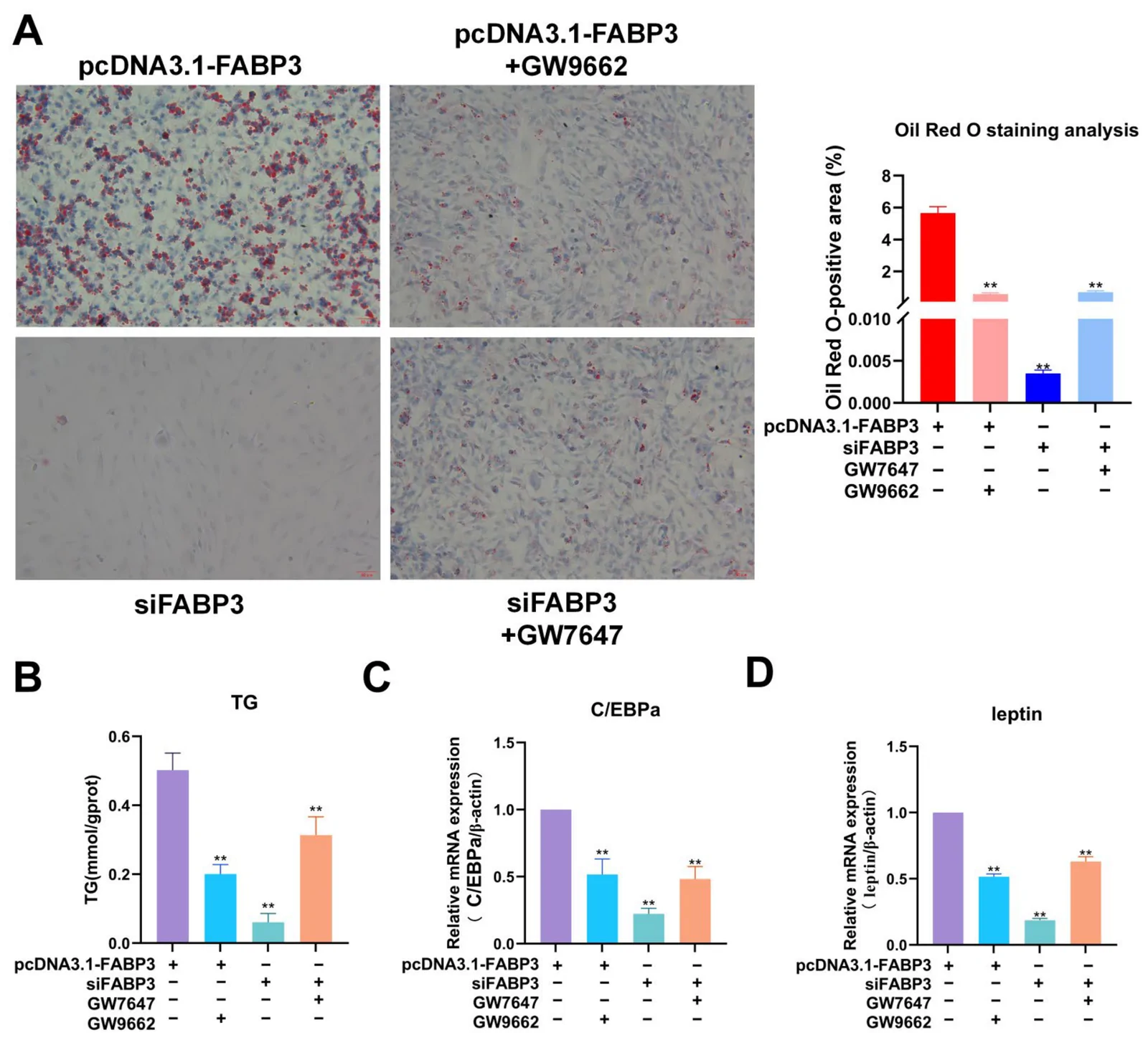
Effect of *FABP3* on PPAR signaling pathway intervention-mediated lipid deposition in chicken primary hepatocytes. (**A**): Oil Red O staining of broiler hepatocytes after GW9662 or GW7647 treatment; the stained area (% Oil Red O-positive) is quantified. (**B**): intracellular TG content in broiler hepatocytes following exposure to GW9662 or GW7647. (**C**,**D**): *C/EBPα* and *leptin* mRNA (each normalized to *β-actin*) in broiler hepatocytes after GW9662 or GW7647 treatment. Significance is denoted as ** *p* < 0.01.

Pharmacological manipulation reproduced the adipocyte findings in hepatocytes. GW9662 trimmed the lipid-positive area of *FABP3*-overexpressing cells, and GW7647 expanded it in *FABP3*-silenced cells (*p* < 0.01 for each; Figure 13A). GW9662 also attenuated *C/EBPα* and *leptin* mRNA (both *p* < 0.01) and TG content (*p* < 0.01) under overexpression, whereas GW7647 augmented all three readouts (*C/EBPα* and *leptin*, both *p* < 0.01; TG, *p* < 0.01) under silencing (Figure 13B–D). *FABP3* therefore modulates lipid deposition in broiler adipocytes and hepatocytes by acting on PPAR signaling.

## 3. Discussion

Abdominal fat deposition is a complex metabolic process driven by multi-tissue crosstalk, in which the liver dominates lipid synthesis and abdominal adipose tissue regulates lipid storage [7]. Rather than being independently controlled by a single gene or pathway, abdominal fat deposition results from synergistic interactions within the fatty acid metabolism regulatory network, which centers on the exogenous uptake, activation, transport, and directed accumulation of fatty acids [7]. The regulatory efficiency of each process collectively determines the level of abdominal fat deposition [7]. Fatty acids released by the hydrolysis of triglycerides are taken up through *CD36* molecule-mediated transport [14]. Fatty acids undergo chain elongation and desaturation catalyzed by ELOVL fatty acid elongase 6 (*ELOVL6*), *SCD* and *SCD5*, yielding fatty acids suited for storage in adipose tissue [15]. *FABP3* takes part in fatty acid transport and promotes expression of lipid metabolism-related genes such as *PPARγ* [16]. In white adipose tissue (WAT), obesity persistently switches on intracellular JAK signaling, and sustained JAK activity interferes with insulin action, aggravating both obesity and insulin resistance [17]. *JAK3*, a member of the JAK kinase family, governs several metabolic processes, including fat accumulation, energy expenditure and insulin sensitivity [18]. Long-chain acyl-CoAs (LCACoAs) generated during fatty acid metabolism also disrupt the insulin signaling pathway [19], and *ACSL4*, an ACSL family member, catalyzes the conversion of long-chain fatty acids to LCACoAs [20]. In the present meta-analysis, mRNA levels of *CD36*, *ELOVL6*, *SCD*, *SCD5*, *ACSL4* and *JAK3* differed between the fat-line/HF and lean-line/LF groups; all six were significantly upregulated in fat tissue and were significantly enriched in lipid metabolism-related pathways. *FABP3* in turn was significantly enriched in lipid metabolism-relevant GO terms, KEGG pathways and GSEA gene sets, marking it as a potential key regulator of this process.

As one of the core members of the fatty acid-binding protein family, *FABP3* is extensively involved in the intracellular uptake, transport, metabolism and signal transduction of fatty acids, serving as a key molecule in the regulatory network of lipid metabolism [21]. Previous studies have preliminarily explored the lipid metabolic regulatory function of FABP3 in mammalian cell models. FABP3 acts not merely as a simple lipid transporter, but may also promote lipid droplet accumulation by upregulating *PPARγ* expression [16], which provides an important theoretical reference for subsequent functional research on *FABP3* in livestock and poultry. *FABP3* exhibits a highly correlated expression pattern with lipogenic marker genes, such as *PPARγ* in porcine adipocytes [22], implying its regulatory role in adipogenesis. *FABP3* overexpression can markedly facilitate lipid droplet formation in 3T3-L1 cells [12], indicating that this gene exerts a positive regulatory effect on lipid droplet biogenesis. Research in chickens has largely centered on preadipocyte differentiation and lipid droplet deposition, and whether *FABP3* takes part in coordinating abdominal fat deposition between the liver and abdominal adipose tissue has not been reported. In the present study, *FABP3* exerted a consistent positive influence on the proliferation and survival of broiler preadipocytes and hepatocytes, indicating that it modulates these processes in both cell types and so supplies a cellular basis for lipid metabolism in the liver and abdominal adipose tissue. *FABP3* raised intracellular triglyceride content and lipid droplet formation in hepatocytes and adipocytes alike, upregulated lipogenic genes such as *C/EBPα* and *leptin*, and promoted the lipid accumulation and differentiation of preadipocytes. That *FABP3* is pro-lipogenic in both hepatic and adipose tissue fits the physiology of broilers, in which the liver dominates lipid synthesis and abdominal adipose tissue handles lipid storage, and it further clarifies how *FABP3* regulates abdominal fat deposition in this species.

Lipid metabolism is governed chiefly by the PPAR signaling pathway, and blocking it markedly suppresses lipogenesis [23], while activating it in chickens strongly promotes adipogenesis [24]. In mammals, FABP3 has been proposed to carry fatty acid ligands into the nucleus, where they bind *PPARγ* and switch on the PPAR pathway to regulate lipid metabolism [25]; whether *FABP3* and the PPAR pathway are directly linked in chickens is, however, still unresolved. Here, in line with the mammalian reports, *FABP3* raised the expression of *PPARγ* and *FABP4*. Because *FABP4* is a classical downstream target of *PPARγ* [26], its altered expression lends further weight to a regulatory action of *FABP3* on the PPAR signaling pathway, raising the possibility that *FABP3* drives downstream lipid-metabolism genes by boosting *PPARγ* expression and, in that way, shapes abdominal fat deposition. The inhibition and agonist-rescue experiments confirmed that the PPAR signaling pathway is an essential mediator of *FABP3*-induced lipid deposition. It should be noted, though, that the two pharmacological agents act on different PPAR isoforms: GW9662 acts as a covalent antagonist of *PPARγ* [23,27], whereas GW7647 is a subtype-selective *PPARα* agonist [28]. The two arms thus demonstrate pathway-level dependency from opposite directions—antagonism blocks the overexpression phenotype and activation restores the silenced phenotype—but on their own they do not establish *PPARγ*-specific signaling. Resolving isoform-specific engagement will require a *PPARγ* agonist (rosiglitazone or ciglitazone) as the rescue arm together with profiling of *PPARα*/*PPARδ* expression.

How *FABP3* and PPAR activation are mechanistically connected likewise calls for cautious reading. The evidence reported here, including the concurrent increase in *PPARγ* and *FABP4* following *FABP3* overexpression and pharmacological intervention at the pathway level, is associative rather than proof of direct activation. Three possibilities have been put forward on the basis of mammalian work: fatty acid ligands may be escorted into the nucleus by FABP3, and there act as activators of *PPARγ* [25]; intracellular fatty acid pools may be remodeled, with a secondary influence on *PPARγ* transcription [21]; or FABP3 and PPARγ may engage in direct protein–protein contact, as proposed for other members of the FABP family [26]. Each of these remains untested in the present system. Establishing which mode operates would require dedicated experiments. These could include a luciferase reporter driven by a PPAR response element, a broader panel of PPARγ targets extending beyond *FABP4*, such as *FASN*, *SCD*, *CD36*, *PLIN1* and *ACC*, or profiling of fatty acid composition.

The consistent upregulation of *FABP3* across all four datasets, together with moderate and non-significant heterogeneity and robust leave-one-out sensitivity, supports *FABP3* as a conserved component of the lipogenic response rather than a line-, diet- or platform-specific marker. This is concordant with the gain- and loss-of-function results showing that *FABP3* is sufficient and necessary, in a PPAR-pathway-dependent manner, for lipid accumulation in primary broiler adipocytes and hepatocytes.

Studies in model organisms have shown that PPARγ and C/EBPα mutually induce each other and that PPARγ activation upregulates the core lipogenic genes *FABP4* and *leptin* [27,28]. Based on this, leptin was used here as a terminal differentiation/lipogenic marker. The avian leptin gene, although recently identified and unusually GC-rich, is conserved among poultry species [29]. In the present study, overexpressing *FABP3* also raised the levels of these key lipogenic genes markedly. The resulting cascade of expression changes ultimately drove proliferation of broiler preadipocytes and hepatocytes together with lipid droplet accumulation and TG synthesis in both cell types. Taken together, we propose that *FABP3* raises the expression of lipid accumulation-related genes and elevates TG levels in adipocytes and hepatocytes via PPAR signaling, thereby enhancing abdominal lipid storage—which may be the mechanism behind the effect of *FABP3* on abdominal fat deposition phenotypes in broilers.

This study has several limitations that should be acknowledged. Every functional assay described above was carried out in vitro with primary cells. Because all functional experiments were performed in vitro in primary cells, no whole-animal validation was included, and a causative contribution of FABP3 to abdominal fat deposition in the intact bird cannot be claimed. The evidence that *FABP3* engages PPAR signaling is indirect and correlative rather than a demonstration of direct activation: the co-upregulation of *PPARγ* and *FABP4* upon *FABP3* overexpression and the pathway-level pharmacological intervention do not establish direct activation, and because the *PPARα* agonist and *PPARγ* antagonist used in the intervention experiments target different isoforms and no direct activation assay (e.g., PPRE-driven reporter) was performed, *PPARγ*-specific signaling remains unresolved. The Oil Red O panels in Figure 6A,B did not separately show the pcDNA3.1 and si*FABP3*-NC controls because the original images were not archived and newly isolated cells did not maintain stable differentiation. However, parallel TG and qPCR analyses included all five groups, and the empty vector and si-NC did not differ from con, so the core conclusion is not affected. Additionally, primary cells were prepared from commercial white-feathered broilers of unspecified breed/strain, so the extent to which the conclusions generalize across genetic backgrounds remains to be determined. Formal purity assessment of primary preadipocytes and hepatocytes was not performed. Flow cytometry for PDGFRα/PREF1 and albumin secretion or CYP3A activity would have been required to exclude contamination with stromal cells or Kupffer cells. Leptin was used as a lipogenic marker, but its biology in birds is atypical. Additional hepatocyte-relevant markers such as *SREBP1c*, *FASN* and *ACC* should be included in future studies. Finally, the meta-analysis integrated datasets generated on different platforms, in different tissues and under different designs (genetic lines and dietary intervention); the AW–Fisher combined *p*-values are nominal, heterogeneity diagnostics were computed in full for the selected candidate but not genome-wide for the complete meta-DEG list, and the per-dataset standard errors used in the heterogeneity analysis were back-calculated from the per-dataset test statistics rather than taken from complete archived standard-error tables. However, 2512 of the 2520 meta-DEGs also pass Benjamini–Hochberg FDR < 0.05, so the meta-DEG list is essentially unchanged by FDR correction. These limitations do not affect the cell-level conclusions but define the boundaries within which they should be interpreted.

## 4. Materials and Methods

### 4.1. Dataset Collection

To minimize the interference of non-genetic factors and ensure statistical power of the analysis results, we established the following inclusion criteria to screen transcriptomic datasets from the NCBI Gene Expression Omnibus (GEO) database: (1) datasets had to be published gene expression profiles of chicken (*Gallus gallus*, domestic broiler) generated using microarray technologies or RNA-Seq; (2) the study design had to include comparable samples from fat and lean groups; (3) all broilers had to be male to control for the potential effect of sex on fat metabolism. Based on these criteria, GSE42980, GSE49121, GSE104038 and GSE104039 were selected for meta-analysis. The species, tissue, design, platform, age and sex information of each dataset is summarized in the Supporting Information, Table S5. The fat line (FL) and lean line (LL) used in GSE42980 were generated by seven generations of divergent selection on abdominal fatness at comparable feed intake and body weight and differed in abdominal fat percentage (AFP) by 2.8-fold at 7 weeks of age [30,31]. This dataset provides RNA-Seq profiles of abdominal fat from 7-week-old males (12 FL, 12 LL) sequenced on an Illumina HiSeq 2000 (https://pubmed.ncbi.nlm.nih.gov/26445145) (accessed on 14 May 2020). GSE49121 was derived from broilers subjected to 30 generations of divergent selection for high (HG) or low (LG) growth, the two lines differing 19.6-fold in AFP and 3.2-fold in body weight at 7 weeks [32]. It provides abdominal fat RNA-Seq data from 7-week-old males (8 HG, 8 LG) (https://www.ncbi.nlm.nih.gov/pubmed/28814270) (accessed on 14 May 2020). GSE104039 is an abdominal fat microarray dataset using an Agilent custom 8 × 60 K chicken gene expression microarray, with males from fat and lean lines cross-classified by diet. The contrast deposited and analysed here compares the HF diet with the LF diet (23 HF, 22 LF). GSE104038 is the liver tissue microarray dataset collected from the same experiment (23 HF, 23 LF) (https://www.ncbi.nlm.nih.gov/pubmed/29514634) (accessed on 22 May 2020) [33]. For both microarray series, probes surviving the filtering steps were mapped to gene symbols, and the resulting symbol lists are provided in the Supporting Information, Tables S9 and S10.

### 4.2. Sequencing Data Preprocessing

Raw reads from GSE42980 and GSE49121 were quality-controlled with fastp (v0.19.7) [34] under its default parameters and workflow. The surviving high-quality reads were then aligned to the chicken reference genome (GCF_016699485.2_bGalGal1.mat.broiler.GRCg7b) with HISAT2 (v2.2.0) [35], again using default parameters. The genome sequence and its annotation file (GCF_016699485.2_bGalGal1.mat.broiler.GRCg7b_genomic.gtf.gz) were obtained from the NCBI genome assembly website (https://ftp.ncbi.nlm.nih.gov/genomes/all/GCF/016/699/485/GCF_016699485.2_bGalGal1.mat.broiler.GRCg7b/) (accessed on 9 October 2025). Postalignment quality was subsequently evaluated using Qualimap (v2.2.2d) [36], and per-gene read counts were quantified using featureCounts (v2.0.1) [37] guided by the same annotation. The two microarray series (GSE104039 and GSE104038), both profiled on Agilent custom 8 × 60 K chicken arrays [33], were processed in R (v4.0.5) with the limma package (v3.60.5) [38]. This workflow applied background correction, quantile normalization and collapsed probes to gene-level expression values, yielding normalized matrices for downstream analyses.

### 4.3. Meta-Analysis of Differentially Expressed Genes (DEGs)

Within each dataset, differential expression was fitted using the algorithm appropriate to its platform: limma (v3.60.5) [38] served the microarray datasets GSE104039 and GSE104038, whereas DESeq2 (v1.36.0) [39], run with default settings, handled the RNA-Seq datasets GSE42980 and GSE49121. Dataset-level *p*-values from the four contrasts were then synthesized into one consolidated statistic per gene with the adaptively weighted Fisher’s method [40]. A gene was declared a significant meta-DEG when its combined *p*-Meta fell below 0.05 and, in addition, its |log_2_FC| reached at least 0.3, it was detected in all four datasets, its direction of change agreed across the contributing datasets, and complete statistics were available everywhere. Because the combined *p*-values are nominal, Benjamini–Hochberg FDR values were additionally computed across all 10,698 tested genes.

### 4.4. Enrichment Analysis of the Meta-DEGs

Functional annotation of the meta-DEGs was carried out in R with clusterProfiler (v4.12.6) [41], querying both the GO and the KEGG resources to clarify how these genes might participate in abdominal fat deposition in broilers. Terms and pathways attaining a *p*-value ≤ 0.05 were treated as statistically significant.

### 4.5. GSEA

GSEA of the meta-DEGs was conducted with the clusterProfiler package (v4.12.6) [41] in R. A gene set was retained as significantly enriched when |Normalized Enrichment Score (NES)| ≥ 1.5 together with *p* < 0.05. The significantly enriched sets were then plotted to further illustrate their expression patterns.

### 4.6. PPI Network Construction

A PPI network covering the meta-DEGs of the enriched pathways was assembled from the Search Tool for the Retrieval of Interacting Genes database (v12.0) (https://string-db.org/) (accessed on 24 January 2026), with the search organism restricted to “*Gallus gallus*”. Interactions with a confidence score above 0.4 were retained, and nodes lacking any connection were hidden. Cytoscape (v3.10.0) [42] served to visualize and interrogate the network, and hub genes were ranked with its built-in Network Analyzer tool, node degree being taken as the indicator of a node’s centrality and functional importance.

### 4.7. Cross-Dataset Assessment of FABP3 Expression, Heterogeneity and Sensitivity

For the selected candidate gene *FABP3*, per-dataset log2FC and *p*-values were extracted from the differential expression results of the four datasets. Per-dataset standard errors (SE) were back-calculated using the formula SE = |log2FC|/z, where z = Φ^−1^(1 − *p*/2), which exactly inverts the Wald tests of DESeq2 and closely approximates the moderated-t tests of limma when residual degrees of freedom are ≥40. For each dataset, the weight was defined as w_i_ = 1/SE_i_^2^. Fixed-effect pooled log2FC was calculated as the inverse-variance weighted mean [43], with pooled SE = (Σw_i_)^(−1/2)^ and 95% confidence interval (CI) = pooled log2FC ± 1.96 × pooled SE. Between-dataset heterogeneity was quantified using Cochran’s Q [44] (chi-square distribution with k − 1 degrees of freedom), I^2^ = max(0, (Q − df)/Q) × 100% [45], and τ^2^ using the DerSimonian–Laird estimator [46]. Random-effects pooled log2FC was computed with weights w_i_* = 1/(SE_i_^2^ + τ^2^), and the corresponding 95% CI was derived. A forest plot was generated using the metafor package (v4.1.1) in R [47]. Leave-one-out sensitivity analyses were performed by iteratively excluding one dataset and recalculating the random-effects pooled log2FC [48]. In addition, an adipose-only analysis excluding the hepatic dataset (GSE104038) was conducted to confirm that tissue differences did not drive the pooled estimate.

### 4.8. Animal Welfare

All procedures involving animals conform to the ARRIVE guidelines (Animal Research: Reporting of In Vivo Experiments) and follow the Guidance on the operation of the Animals (Scientific Procedures) Act 1986, together with EU Directive 2010/63 on the protection of animals used for scientific purposes. The Animal Ethics Committee of Northeast Agricultural University approved the experimental protocol (Approval No.: NEAUEC20230209), and every effort was made to relieve animal suffering.

### 4.9. Primary Chicken Preadipocyte Isolation and Culture

Donor birds of this age were chosen because preadipocytes obtained before rapid abdominal fat accretion begins still possess strong proliferative and adipogenic capacity [49]. Abdominal fat tissue was harvested from male 2–3-week-old commercial white-feathered broilers provided by Lanxi Yijia Agriculture and Animal Husbandry Co., Ltd., (Lanxi, Heilongjiang, China)., rinsed with sterile PBS (Biosharp, Labgic Technology Co., Ltd., Beijing, China) and dissected to remove blood vessels and fascia. For each biological replicate, preadipocytes were isolated from 5 donor broilers, and three independent replicates were performed, using a total of 15 donor broilers. Minced tissue was digested with 0.2% type I collagenase (Sigma-Aldrich, Inc., St. Louis, MO, USA) at 37 °C for 60 min under continuous oscillation, with mixing every 5 min [50]. Culture medium was added to halt the reaction, and the digest was passed successively through 100-mesh and 600-mesh screens before a 10 min at 2000× *g* on a refrigerated centrifuge (Heal Force Bio-meditech Holdings Limited, Shanghai, China). The pellet was then treated with red blood cell lysis solution for 10 min; after one further centrifugation, it was washed 1–2 times in culture medium, and the resulting suspension was plated at 1 × 10^5^ cells/cm^2^ and kept at 37 °C under 5% CO_2_. Purity was inferred from the fibroblast-like morphology of the differentiating cells and from the uniform cytoplasmic lipid filling observed after induction.

### 4.10. Primary Chicken Hepatocyte Isolation and Culture

Livers were collected from 3-week-old male commercial white-feathered broilers supplied by Lanxi Yijia Agriculture and Animal Husbandry Co., Ltd (Lanxi, Heilongjiang, China). [51], with one donor per biological replicate and three replicates in total (3 donors). Donors were fasted for 3–6 h with water freely available, anesthetized by intraperitoneal injection of 50 mg/kg sodium pentobarbital, and given 1500–2000 IU/kg heparin sodium through the wing vein. Perfusion solutions A, B and C, along with prewarmed basal medium containing 10% FBS and antibiotics, were prepared in advance. After supine fixation, abdominal disinfection and laparotomy, a cannula was placed in the portal vein, and the inferior vena cava acted as the outflow channel. Solution A was delivered at 20–30 mL/min for 10–15 min until the liver blanched; after a brief rinse with Solution B, collagenase-containing Solution C was recirculated at 15–20 mL/min for 15–20 min until the organ softened and showed surface cracks. The digested liver was dispersed in cold serum-containing medium, sieved through 100- and 200-mesh filters, and pelleted by low-speed centrifugation at 4 °C and 50× *g*, followed by 2–3 cycles of washing. Hepatocyte identity was confirmed by the typical polygonal morphology and uniform albumin-positive staining expected of primary chicken hepatocytes.

### 4.11. Transfection of Primary Chicken Preadipocytes and Hepatocytes and Induction of Differentiation

Preadipocytes and hepatocytes were plated in 12-well plates (1 × 10^5^ cells/well) and transfected at 50–60% confluence with the *FABP3* overexpression vector (pcDNA3.1-*FABP3*), small interfering RNA (siRNA) or their matched negative controls using Lipo6000™ (Beyotime Biotechnology, Shanghai, China); qPCR and WB analysis verified the transfection efficiency. Where indicated, the *PPARγ* antagonist GW9662 (Dalian Meilun Biotechnology Co., Ltd., Dalian, Liaoning, China) or the *PPARα* agonist GW7647 (Dalian Meilun Biotechnology Co., Ltd., Dalian, Liaoning, China) was administered 2 h before delivery of the *FABP3* overexpression vector and siRNA, respectively. Differentiation was initiated 24 h after transfection by switching to differentiation medium supplemented with 200 μmol/L oleic acid (Wuhan Yuansheng Biotechnology Co., Ltd., Wuhan, Hubei, China) [49]. The sequences of siFABP3 and pcDNA3.1-*FABP3* are listed in Table 1.

### 4.12. CCK-8 Viability Assay

For viability measurements, transfected cells were transferred to 96-well plates (5 × 10^4^ cells/well, 100 μL/well). After 72 h of culture, each well received 10 μL of CCK-8 reagent (Dalian Meilun Biotechnology Co., Ltd., Dalian, Liaoning, China) and plates were held at 37 °C under 5% CO_2_ for 3 h [52] before absorbance readings at 450 nm on a microplate reader. Viability was assessed with CCK-8. It is presented either as blank-corrected absorbance at 450 nm, that is, OD450 (sample) − OD450 (blank); or normalized to the control group as (OD_sample − OD_blank)/(OD_control − OD_blank) × 100%. Cell viability was expressed in these two forms as specified in the corresponding figure legends. Six technical wells per group were averaged within each biological replicate prior to statistical analysis.

### 4.13. EdU Proliferation Assay

A parallel set of transfected cells grown on coverslips in 24-well plates (3 × 10^5^ cells/well) was used for EdU labeling. EdU working solution (Beyotime Biotechnology, Shanghai, China) was applied for 2 h at 48 h after plasmid transfection or 72 h after siRNA transfection [52]. Cells were then fixed in 4% paraformaldehyde(Wuhan Boerfu Biotechnology Co., Ltd., Wuhan, Hubei, China) for 15 min and processed through the Click reaction. Labeled cells were examined under a fluorescence microscope (Nikon Corporation, Tokyo, Japan; excitation 495 nm, emission 519 nm). The proliferation index was defined as the number of EdU-positive nuclei divided by the total number of DAPI-stained nuclei per field, expressed as a percentage. Three random fields per coverslip were scored and averaged to yield a single value per biological replicate, and all experiments were repeated three times with cells from different donor broilers.

### 4.14. RNA Extraction and qPCR

Total RNA was isolated with a total RNA extraction kit (Beijing Solarbio Science & Technology Co., Ltd., Beijing, China), and RNA concentration and purity were read on a microvolume spectrophotometer (Shanghai Jiapeng Technology Co., Ltd., Shanghai, China). cDNA was then generated by reverse transcription. Each 20 μL qPCR reaction contained 10 μL PerfectStart^®^ Green qPCR SuperMix(TransGen Biotech Co., Ltd., Beijing, China), 1 μL each of forward and reverse primer, 2 μL cDNA and nuclease-free water to volume. Amplification started at 94 °C for 2 min and proceeded through 45 cycles of 94 °C for 5 s and 60 °C for 30 s, followed by melting-curve analysis from 65 to 95 °C with 0.5 °C increments every 5 s. *β-actin* served as the reference gene, and relative expression of each target was computed with the 2^−∆∆CT^ method [53]. The primer sequences used for qPCR are given in Table 1.

### 4.15. Western Blotting

Samples were lysed on ice for 15 min in Radio-Immunoprecipitation Assay (RIPA) lysis buffer(Wuhan Boerfu Biotechnology Co., Ltd., Wuhan, Hubei, China) supplemented with protease and phosphatase inhibitors (Dalian Meilun Biotechnology Co., Ltd., Dalian, Liaoning 116600, China), and protein amounts were quantified with the Bicinchoninic acid (BCA) assay. Proteins were resolved on 12% SDS-polyacrylamide gels and blotted onto polyvinylidene fluoride membranes (MilliporeSigma, Burlington, MA, USA) [54]. After blocking in 5% bovine serum albumin(Dalian Meilun Biotechnology Co., Ltd., Dalian, Liaoning, China) for 60 min at 37 °C, membranes were probed overnight at 4 °C under gentle shaking with the primary antibodies anti-FABP3, anti-FABP4, anti-PPARγ (Proteintech Group, Inc., Rosemont, IL, USA) and anti-β-actin (ABclonal Technology Co., Ltd., Wuhan, Hubei, China), and then exposed to horseradish peroxidase-labeled secondary antibodies (Jackson ImmunoResearch Laboratories, Inc., West Grove, PA, USA) for 1 h at room temperature. Chemiluminescent reagent revealed the bands, whose relative gray values were quantified in Image-Pro Plus 6.0 software (Media Cybernetics, Inc., Rockville, MD, USA).

### 4.16. Oil Red O Staining

Cells were collected at 24, 48, 72 and 96 h after the induction of differentiation and fixed in 4% paraformaldehyde for 15 min. Oil Red O working solution was applied in the dark for 8 to 10 min, after which excess dye was removed with 75% isopropanol (Sinopharm Chemical Reagent Co., Ltd., Shanghai, China), and the samples were rinsed in distilled water. Nuclei were counterstained with hematoxylin for 3–5 min, followed by differentiation, bluing and mounting, and the preparations were imaged under a light microscope (Nikon Corporation, Tokyo, Japan). For quantification, retained dye was eluted in 100% isopropanol for 10 min, and absorbance was read at 510 nm on a microplate reader (Molecular Devices, LLC, San Jose, CA, USA) [55]. In parallel, image-based quantification in Image-Pro Plus 6.0 applied a uniform red-channel threshold to all images within an experiment, and results are reported as the percentage of Oil Red O-positive area relative to the total field area.

### 4.17. Triglyceride Quantification

Cell lysates were assayed for TG concentration through enzymatic conversion to quinoneimine in the presence of peroxidase, and values were interpolated from a standard curve built with known TG standards. Protein content of an identical lysate aliquot was measured with a BCA Protein Assay Kit (Beijing Solarbio Science & Technology Co., Ltd., Beijing, China) for normalization, so that the final TG content was expressed as the ratio of TG concentration to protein concentration [56].

### 4.18. Statistics

Reliability and reproducibility were safeguarded by running every cell-culture experiment three times independently, each biological replicate deriving from a separate cell preparation; technical replicates were averaged within each biological replicate before analysis. GraphPad Prism 11.0 served for data visualization and statistical analysis. Multiple treatment groups were compared against the control group (con) with one-way analysis of variance (one-way ANOVA) followed by Dunnett’s post hoc test. The predefined pairwise contrasts—pcDNA3.1-*FABP3* versus pcDNA3.1-*FABP3* + GW9662 and si*FABP3* versus si*FABP3* + GW7647—were assessed with unpaired two-tailed Student’s t tests. All measurement data are expressed as mean ± standard error of the mean (mean ± SEM), and *p* < 0.05 was taken as the threshold for statistical significance.

## 5. Conclusions

By meta-analysing abdominal fat and liver transcriptomic datasets, the present work identified 2520 meta-DEGs, significantly enriched in lipid metabolism pathways such as PPAR signaling. *FABP3* recurred in the GO, KEGG and GSEA analyses and was therefore selected for in vitro functional validation. *FABP3* enhanced the proliferation and survival of primary broiler preadipocytes and hepatocytes, raised triglyceride content and lipid droplet formation in both cell types, raised the lipogenic markers *C/EBPα* and *leptin*, and drove both lipid accumulation and preadipocyte differentiation. It also upregulated *PPARγ* and *FABP4*, and the pathway perturbation experiments demonstrated that the pro-lipogenic action of *FABP3* depends on PPAR signaling pathway activity. These results place *FABP3* as a candidate regulator of lipid deposition in broiler liver and abdominal adipose tissue and provide a starting point for dissecting the liver-adipose axis that governs abdominal fat deposition.

## Figures and Tables

**Table 1:** Primer, siFABP3 and pcDNA3.1-*FABP3* sequences.

Primer Name	Accession Number	Forward /Reverse	Primer Sequence (5′ to 3′)	Purpose
*FABP3* siRNA-1	-	F	GCGACAAGGUGACGGUGAATT	silencing of FABP3 (sense/antisense strands)
		R	UUCACCGUCACCUUGUCGCTT	
*FABP3* siRNA-2	-	F	GGAAGUUGAUUCUGACUCUTT	silencing of FABP3 (sense/antisense strands)
		R	AGAGUCAGAAUCAACUUCCTT	
*FABP3* siRNA-3	-	F	CCAAUUUCGAUGAGUACAUTT	silencing of FABP3 (sense/antisense strands)
		R	AUGUACUCAUCGAAAUUGGTT	
si*FABP3*-NC	-	F	UUCUCCGAACGUGUCACGUTT	non-targeting control
		R	ACGUGACACGUUCGGAGAATT	
pcDNA3.1-*FABP3*	-	F	GCTAGCGCCACCATGGTGGAAGCG	cloning primers for the overexpression construct (NheI/XhoI sites); the construct was confirmed by Sanger sequencing with universal vector primers
		R	CTCGAGCTATGATGCCTTCTCATAGGTGCG	
Gallus gallus *β-actin*	NM_205518.2	F	GTGGATCAGCAAGCAGGAGT	reference gene for qPCR normalization
		R	ATCCTGAGTCAAGCGCCAAA	
Gallus gallus *C/EBPa*	NM_001031459.2	F	TCCCACCTGCAGTACCAGAT	qPCR detection of C/EBPα
		R	GCGGTACTCGTTGCTGTTCT	
Gallus gallus *leptin*	NM_001007958.2	F	GCAACGATTGAGGCGATTCC	qPCR detection of leptin
		R	CCAGCAGCTCCTTCAACTCA	
Gallus gallus *FABP3*	NM_001030889.2	F	AAGGAGACATCGCTAGTGCG	qPCR detection of FABP3
		R	ATGCCTTCTCATAGGTGCGG	
Gallus gallus *PPARγ*	NM_001001460.2	F	CATCAGGTTTGGGCGAATGC	qPCR detection of PPARγ
		R	TAACTGGTCGATGTCGCTGG	
Gallus gallus *FABP4*	NM_204290.2	F	GCCTGACAAAATGTGCGACC	qPCR detection of FABP4
		R	ATTAGGCTTGGCCACACCAG	

## Data Availability

The datasets GSE42980, GSE49121, GSE104039, and GSE104038 analyzed in this study are publicly available in the NCBI Gene Expression Omnibus (GEO) database (https://www.ncbi.nlm.nih.gov/geo/, accessed on 14 May 2020).

## Supplementary Materials

The following supporting information can be downloaded at: https://www.mdpi.com/article/10.3390/ijms27219413/s1.

## Abbreviations

DEGs	differentially expressed genes
RNA-Seq	RNA sequencing
FABP3	fatty acid-binding protein 3
GSEA	Gene set enrichment analysis
KEGG	Kyoto Encyclopedia of Genes and Genomes
GO	Gene Ontology
CCK-8	cell counting kit-8
TG	triglyceride
C/EBPα	CCAAT/enhancer-binding protein α
qPCR	quantitative real-time polymerase chain reaction
WB	Western blot
LPL	lipoprotein lipase
PPI	protein–protein interaction
BP	biological process
CC	cellular component
MF	molecular function
ACACA	acetyl-CoA carboxylase alpha
ELOVL6	ELOVL fatty acid elongase 6
PPAR	peroxisome proliferator-activated receptor
FASN	fatty acid synthase
SCD	stearoyl-CoA desaturase
FDR	false discovery rate (adjusted *p*-value)
SEM	standard error of the mean
PPARγ	peroxisome proliferator-activated receptor gamma
FABP4	fatty acid-binding protein 4
FABP	fatty acid-binding proteins
FL	fat line
LL	lean line
AFP	abdominal fat percentage
HG	high growth
LG	low growth
SiRNA	small interfering RNA
SCD5	stearoyl-CoA desaturase 5
RIPA	radio-immunoprecipitation assay
NC	negative control
EDU	5-Ethynyl-2′-deoxyuridine
log_2_FC	log_2_(fold change)
